# Towards diverse agricultural land uses: socio-ecological implications of European agricultural pathways for a Swiss orchard region

**DOI:** 10.1007/s10113-023-02092-5

**Published:** 2023-07-22

**Authors:** Takamasa Nishizawa, Sonja Kay, Johannes Schuler, Noëlle Klein, Tobias Conradt, Michael Mielewczik, Felix Herzog, Joachim Aurbacher, Peter Zander

**Affiliations:** 1 Farm Economics and Ecosystem Services, Leibniz Centre for Agricultural Landscape Research (ZALF) e.V., Müncheberg, Germany; 2 Agricultural Landscapes and Biodiversity, Agroscope, Zurich, Switzerland; 3 Planning of Landscape and Urban Systems (PLUS), ETH Zürich, Zürich, Switzerland; 4 Potsdam Institute for Climate Impact Research, Potsdam, Germany; 5 Department of Sustainability Assessment and Agricultural Management, Socioeconomics, Agroscope, Ettenhausen, Switzerland; 6 Institute of Farm and Agribusiness Management, Justus-Liebig-University Giessen, Giessen, Germany

**Keywords:** Global land use drivers, Shared socioeconomic pathways (SSPs), Climate change, Scenario development, Diverse agricultural land uses, Trade-off

## Abstract

Diverse agricultural land uses are a typical feature of multifunctional landscapes. The uncertain change in the drivers of global land use, such as climate, market and policy technology and demography, challenges the long-term management of agricultural diversification. As these global drivers also affect smaller scales, it is important to capture the traits of regionally specific farm activities to facilitate adaptation to change. By downscaling European shared socioeconomic pathways (SSPs) for agricultural and food systems, combined with representative concentration pathways (RCP) to regionally specific, alternative socioeconomic and climate scenarios, the present study explores the major impacts of the drivers of global land use on regional agriculture by simulating farm-level decisions and identifies the socio-ecological implications for promoting diverse agricultural landscapes in 2050. A hilly orchard region in northern Switzerland was chosen as a case study to represent the multifunctional nature of Swiss agriculture. Results show that the different regionalised pathways lead to contrasting impacts on orchard meadows, production levels and biodiversity. Increased financial support for ecological measures, adequate farm labour supplies for more labour-intensive farming and consumer preferences that favour local farm produce can offset the negative impacts of climate change and commodity prices and contribute to agricultural diversification and farmland biodiversity. However, these conditions also caused a significant decline in farm production levels. This study suggests that considering a broader set of land use drivers beyond direct payments, while acknowledging potential trade-offs and diverse impacts across different farm types, is required to effectively manage and sustain diversified agricultural landscapes in the long run.

**Supplementary information:**

The online version contains supplementary material available at 10.1007/s10113-023-02092-5.

## Introduction

Agriculturally diversified land uses are closely linked to multifunctional landscapes (Frei et al. 2020; Hölting et al. 2020). These diverse land uses can contribute to the enhanced delivery of wide-ranging ecosystem services (e.g. biomass, biodiversity, aesthetic values, the quality of soil, air, water) (Albert et al. 2017; Dou et al. 2021). Understanding the drivers of agricultural land use changes (LUCs) and their effects is essential for the successful management of more diversified, heterogeneous agricultural landscapes. However, this task is complex due to the uncertain future pathways of agricultural transformation driven by today’s fast-paced world. Agricultural LUCs can be triggered by distinct combinations of land use drivers rather than single key drivers (van Vliet et al. 2015). The global drivers that affect agricultural land use include market and labour conditions (e.g. price volatility, changes in workforce), technology (e.g. digitalisation and mechanisation), demography (e.g. population growth, altered age structure), consumption changes (e.g. less demand for meat), policies (e.g. more restrictive agri-environmental policies) and climate change (e.g. extreme weather events) (Alexander et al. 2015; Pröbstl-Haider et al. 2016; Stehfest et al. 2019). These drivers are likely to continue to change over the next few decades (Bryan et al. 2016; Masson-Delmotte et al. 2019).

The effects of global drivers inevitably manifest themselves at smaller scales, such as the regional and farm level (Fronzek et al. 2019; Arnalte-Mur et al. 2020). The majority of European agricultural regions and systems is highly dependent on the path of global drivers (Debonne et al. 2022), and as a result, farming activities constantly adapt to global drivers (Stürck et al. 2018) to survive in today’s competitive, market-oriented agricultural sector (Nybom et al. 2021). Farmers’ decisions regarding farm activities and land management intensity are influenced by a variety of factors, some of which are stable (topography, soil characteristics) while others change at various paces across different regions (Fronzek et al. 2019; Levers et al. 2018); these factors include crop profitability, regulations, agri-environmental measures, farm technology, climate, farm labour supply and food consumption patterns. Thus, in the long term, we can expect major changes to evolve in agricultural land use (Valbuena et al. 2010; Popp et al. 2017; Stehfest et al. 2019). As these drivers continue to shape agricultural landscapes, understanding how global drivers influence farmers’ decisions becomes crucial (van Vliet et al. 2015). To enhance the diverse benefits provided by agricultural landscapes, however, potential trade-offs must be acknowledged, as various benefits react to changes differently (Beckmann et al. 2019; Botzas-Coluni et al. 2021). Scaling up from the regional level to the national level, the knowledge of farmers’ adaptation decisions to future uncertain changes can facilitate the development of food and agri-environmental policies that consider the differences in needs across regional and local levels (Schaldach et al. 2011; Bauer & Steurer 2014). However, there is a lack of studies assessing the impacts of global drivers on European agriculture at the regional level (Debonne et al. 2022).

A comparative scenario approach is a prominent method for addressing future uncertainty in changing drivers (Vervoort et al. 2014; Von Lampe et al. 2014; Riahi et al. 2017) as well as scale issues (i.e. straddling impacts across spatial scales) (O’Neill et al. 2020; Stratigea & Giaoutzi 2012). The shared socioeconomic pathways (SSPs), as described by O’Neill et al. (2014, 2017), offer a consistent set of scenarios based on a globally accepted framework. The SSPs encompass five contrasting narratives that describe how socioeconomic factors could change, including demographics, economic growth, education, urbanisation and the rate of technological development over the next century. Combining different levels of greenhouse gases described in the representative concentration pathways (RCPs) allows us to evaluate the impacts of both climate change and the change in socioeconomic drivers under the same scenario framework (Meinshausen et al. 2011; van Vuuren et al. 2011). In a recent study, Mitter et al. (2020) developed Eur-Agri-SSPs (European-Agricultural-SSPs) by adapting the global scale SSPs to suit the context of the European agriculture and food sector. The narratives of Eur-Agri-SSPs capture the uncertainty of the following five socioeconomic, technological and environmental drivers: (1) population and urbanisation; (2) economy and markets; (3) policies and institutions; (4) technologies and (5) environmental and natural resources. Similarly, Lehtonen et al. (2021) extended SSPs to a national scale and applied them to the agricultural and food sectors in Finland, while Pedde et al. (2021) developed a set of multi-driver SSPs for the UK. Each of these studies integrated local and national knowledge with top-down insights derived from the global SSPs. Mitter et al. (2020) and Pedde et al. (2021) propose further SSP extensions towards smaller scales, while Prost et al. (2023) emphasise the importance of explicitly incorporating the farm level into future-oriented studies to support farm transition.

We chose a rural orchard region in northern Switzerland for this study. This region serves as a representative case, as it is not only a small-scale region but also features a combination of different agricultural land uses and farm types. Traditional fruit orchards, a characteristic element, illustrate the current multifunctional nature of agriculture in the region. This study then aims to explore the major impacts of future global land use drivers on regional agricultural landscapes that are shaped by farm-level decisions to obtain a better understanding of the regionally specific socio-ecological implications for diversified agricultural landscapes in the long term. To achieve this objective, we propose an integrated model-based scenario approach: we downscale the Eur-Agri-SSPs and RCPs to create regionally tailored scenarios. In this process, we consider the actual Swiss policy agendas and initiatives (cf, Finger 2021; Schweizerischer Bundesrat 2022). The scenarios were implemented in the integrated Land Use Change and Impact Assessment model (LUCIA) (Nishizawa et al. 2022) to simulate agricultural LUCs at the farm level. Our modelling approach includes the full set of farming activities across different farm types. This is crucial to consider, as the decisions made on farms vary depending on their characteristics (Huber et al. 2023) and the development of indicators for the assessment of farms’ environmental performances is also being driven by the Swiss government (Mann & Kaiser 2023). This approach allows us to investigate how unique regional traits and farm heterogeneity can be included in future LUCs and to identify trade-offs in the socio-ecological system by considering different climate and socioeconomic conditions in different future scenarios. Based on the above considerations, we develop the following research questions:

1. What are the major impacts of global socioeconomic and climate conditions on future regional agricultural land use, given the farming characteristics of the case study region?
2. Based on these outcomes, what trade-offs are observed in the socio-ecological system of agricultural land use?
3. What regionally specific socio-ecological implications can we gain to promote diverse agricultural landscapes in 2050 given the resulting agricultural land use changes?

## Methods and materials

### Case study region

**Fig. 1:**
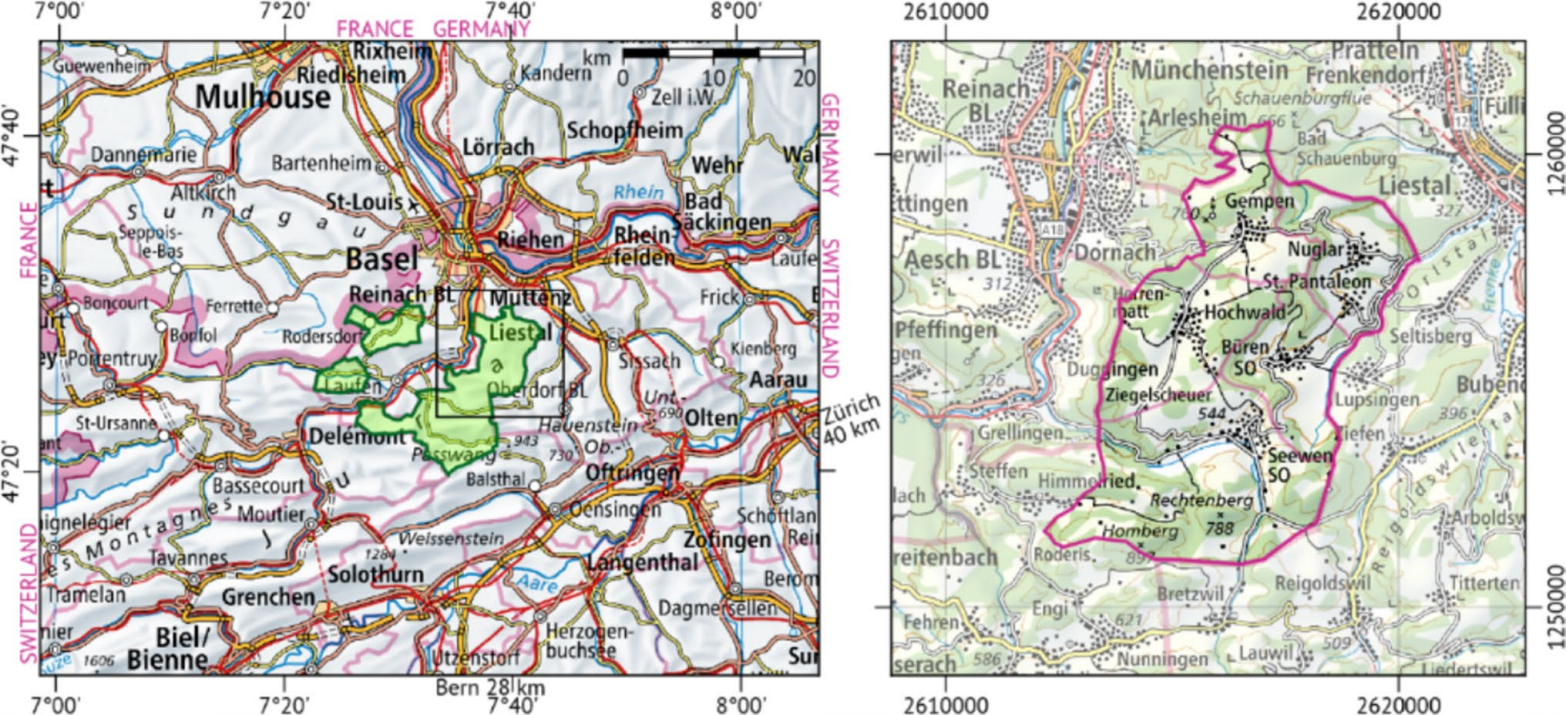
Location of the Schwarzbubenland (left) and the five municipalities considered in this study (right). The square on the left map highlights the extent of the magnified area shown on the right map. The coordinates and grid lines of the right map refer to the Swiss national grid with a grid line spacing of 10 km. Base maps © 2022 Federal Office of Topography Swisstopo

The study region is located in the eastern part of Schwarzbubenland (SBL), which is a part of the Swiss canton of Solothurn in northern Switzerland (Fig. 1). The region covers an area of 42.2 km^2^ and is home to 74 farms, each with an average size of approximately 24 ha. This rural area represents a characteristic multifunctional agricultural landscape, predominantly shaped by traditional agroforestry (ALW 2020). The regional agroforestry consists of *Streuobstwiesen*, high-stem cherry orchards of scattered fruit trees combined with perennial grasslands, which are grazed by cattle and/or mown regularly for fodder production (Herzog 1998). These orchard meadows are associated with agro-biodiversity hotspots (Kay et al. 2018), which have the potential to increase the species richness of habitats in rural agricultural landscapes (Horak et al. 2013). However, due to higher labour costs, recent infestations by invasive fruit flies and limited specific protection measures, many farms tend to drop cherry production from their business portfolio. Given the current situation, the ability of these farms to maintain such traditional multifunctional agricultural landscapes has become increasingly uncertain. Arable land accounts for only 32% of the farmland (ALW 2020).

### Overall approach

**Fig. 2:**
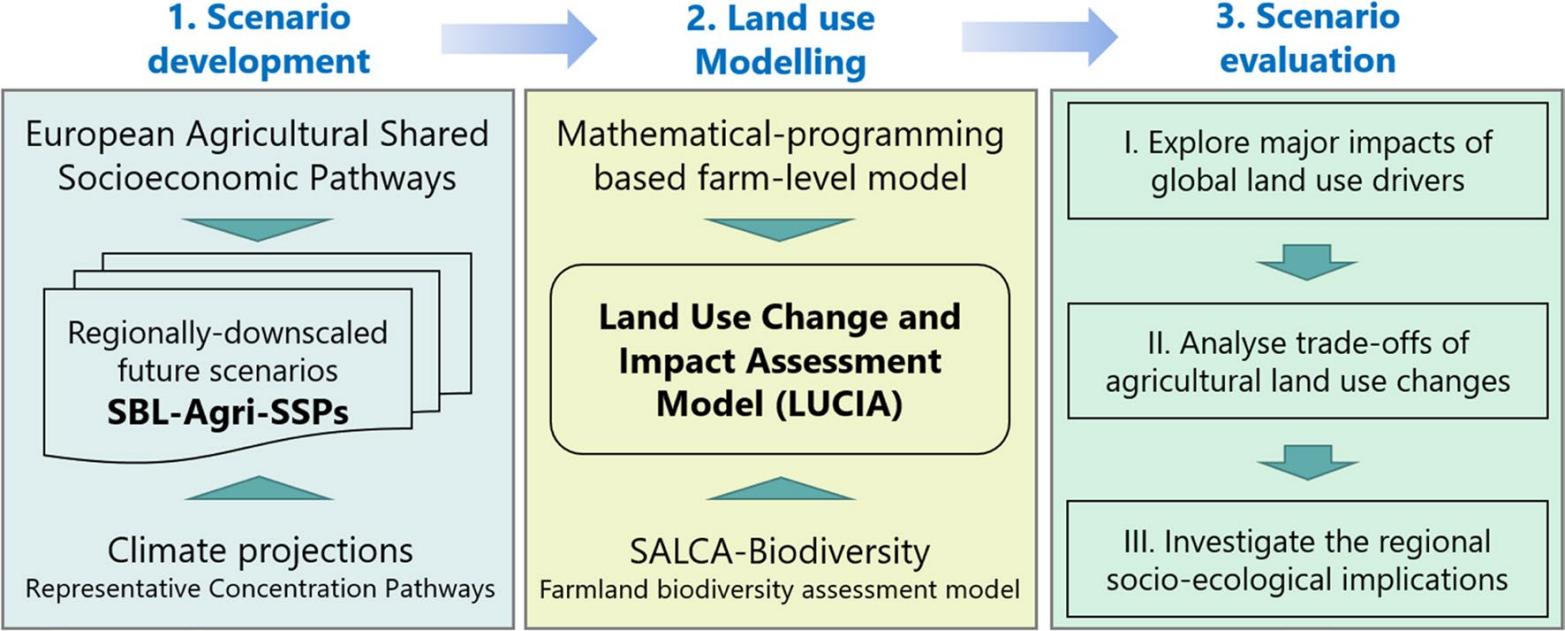
Framework of an integrated model-based scenario approach for evaluating the impacts of climate change and socioeconomic developments on future agricultural land use at the regional scale (SBL-Agri-SSPs = Schwarzbubenland agricultural shared socioeconomic pathways)

We developed an integrated model-based scenario approach, which consists of three steps (Fig. 2). First, we derived future scenarios for the regional agriculture and food sector in 2050, called the SBL agricultural socioeconomic pathways (SBL-Agri-SSPs) scenarios, which were downscaled from the Eur-Agri-SSPs (Mitter et al. 2020) capturing the regional trends and traits of socioeconomic and climate conditions for agricultural land use. To account for climate impacts, these scenarios were combined with climate projections that are consistent with RCPs. Second, the relevant components of SBL-Agri-SSPs were parameterised for the LUCIA (Nishizawa et al. 2022) to simulate agricultural LUCs at the farm level. Third, we evaluated their corresponding impacts on the socioeconomic system of agricultural land use. In doing so, we explored the most significant impacts of the SBL-Agri-SSPs, analysed the trade-offs and discussed the implications of the resulting changes in agricultural land use for promoting diverse agricultural landscapes in the future.

### Climate change impact

Climate change has direct and indirect impacts on agricultural land use and production (Olesen & Bindi 2002; Nelson et al. 2014): First, yields change due to changes in air temperature, precipitation, solar radiation, etc. Second, farmers adapt to climate change by developing new cropping systems with modified shares of common or new crops. To capture the first aspect, we used bias-adjusted EURO-CORDEX (12.5-km grid resolution) climate scenarios for the RCP4.5 and 8.5 emission pathways. Each grid cell has up to 33 parallel realisations of daily meteorological parameters (air temperature, precipitation, solar radiation, wind speed, etc.) for each scenario. These climate scenario data were used in the novel crop yield model ABSOLUT (Conradt 2022). CO^2^ fertilisation or changes in management were not represented. While simulated climate change trends in yield levels were clear, trends regarding yield risks (variations) could not be detected between 2020 and 2050 or between RCP4.5 and RCP8.5. Hence, the relative yield changes were included in the LUCIA, but not the yield risks. To capture the adoption of new crops as a climate adaptation strategy, we identified a so-called climate analogue region for the case study region, following Mahony et al. (2017). Utilizing this method, we identified soy, sunflowers and grain maize as potential crops likely to be adopted and cultivated in the study region by 2050. Due to a lack of data, soy was not included in the yield simulation. Therefore, we applied the same relative yield change from sunflower to soy, as they are both oilseed crops (for a more detailed explanation of the methodology and results, please refer to Online Resource 1).

### Development of SBL-Agri-SSP scenarios

To cover a wide range of possible agricultural land use scenarios for 2050, we selected three contrasting scenarios from the Eur-Agri-SSPs (Mitter et al. 2020): Eur-Agri-SSP1 (agriculture on sustainable paths), Eur-Agri-SSP2 (agriculture on established paths) and Eur-Agri-SSP5 (agriculture on high-tech paths). Each of these scenarios was individually translated to the study region one by one to construct regionally tailored SBL-Agri-SSP scenarios that capture the regional agricultural traits (biophysical conditions, farm management and structures, and farm heterogeneity), in addition to socioeconomic changes (population and urbanisation, economy and markets, policies and institutions, technologies, and environmental and natural resources) for the region. The narratives of the developed SBL-Agri-SSPs were then parameterised, including the reference scenario that was based on the current data for 2020. To combine SBL-Agri-SSPs with the selected RCPs for this study, we referred to O’Neill et al. (2020) and Hausfather and Peters (2020). The highest forcing pathway, RCP8.5, according to O’Neill et al. (2020), is only likely to occur following the fossil fuel development pathway (the global SSP5). Hausfather and Peters (2020) also argued that the business-as-usual scenario is unlikely to follow RCP8.5. Therefore, we opted for more plausible combinations of the SBL-Agri-SSP1 and SSP2 with RCP4.5. The SBL-Agri-SSP5 was combined with RCP8.5 because we assume that society as a whole will make more mitigation efforts in the SBL-Agri-SSP1 and 2 than in the SBL-Agri-SSP5 (see also O’Neill et al. 2020). Below, we summarise the key outcomes of the scenario development for each SBL-Agri-SSP scenario. Table 1 gives an overview of the SBL-Agri-SSPs, and Table 2 provides the relative change in the key parameters in the LUCIA. More detailed and quantitative descriptions are provided in Online Resource 2. Notably, this study does not account for the effects of inflation.

**Table 1:** Summary of the developed future scenarios, the SBL-Agri-SSPs. A more detailed description of the scenarios is given in Online Resource 2

Key characteristics	Reference scenario (Ref)		SBL-Agri-SSP1 (Regional agriculture on the organic sustainable path)		SBL-Agri-SSP2 (Regional agriculture on the BAU scenario path)		SBL-Agri-SSP5 (Regional agriculture on the liberalised path)	
Temporal scale	2020		2050		2050		2050	
Socioeconomic condition	Current observation		Eur-Agri-SSP1		Eur-Agri-SSP2		Eur-Agri-SSP5	
Climate scenario	-		RCP 4.5		RCP 4.5		RCP 8.5	
Farm structure	Current status (avg. farm size 24 ha)		More smaller farms with smaller fields (avg. 23 ha)		Lower number of farms (avg. 27 ha)		Almost only large-scale farms with livestock (avg. 60 ha)	
Farm types (weight)	Small dairy farm Large dairy farm Suckler farm Orchard farm Small farm	(12 %) (30 %) (32 %) (14 %) (12 %)	Small dairy farm Large dairy farm Suckler farm Orchard farm Small farm	(11 %) (16 %) (35 %) (25 %) (13 %)	Small dairy farm Large dairy farm Suckler farm Orchard farm Small farm	(10 %) (28 %) (37 %) (14 %) (11 %)	Large dairy farm Suckler farm Orchard farm	(55 %) (39 %) (6 %)
Crop production	· Both for fodder production and cereals for human consumption		· No synthetic chemical l fertilisers/plant protection products · Only clover allowed for fodder production		· Same as the reference		· Intensive use of mechanisation for larger field size · Eased crop rotation system	
New crops	-		·Sunflower, soy, and grain maize (cash crops) ·Clover-grass (fodder)		·Sunflower and grain maize (cash crops) ·Soy (fodder)		·Sunflower and grain maize (cash crops) ·Soy (fodder)	
Grassland	· Primarily extensive grass production (meadow/pasture)		· Intensive management still possible · But only manure allowed		· Same as the reference		· No pasture · Used exclusively for hay production	
Orchards	· Orchards combined with extensive grass production · Non-commercial fruit production		· Commercial fruit production sold to regional markets		· Same system as the reference		· Same system as the reference but without subsidies	
Livestock production & Fodder system	· Primarily free-range · Low livestock intensity ·Grass-based fodder system * except for large dairy farms		· Smaller scale · Fed exclusively roughage (low milk performance)		· Higher livestock intensity with mechanisation . Same fodder systems as the reference		· Kept in large cattle barns with high livestock intensity · No grazing and no restriction for feed (high milk performance)	
Biodiversity measures	· 7% of EFAs on farmland		· 15% of EFAs on farmland · 10% of EFAs on arable land		· 10% of EFAs on farmland · 4% of EFAs on arable land		· No regulation	

*EFA* ecological focused area

*At least 75% of feed must be roughage and the proportion of concentrates (cereals) must be less than 10%

**Table 2:** Relative change of key parameters under each future scenario in comparison to the current level (100%). The parameter changes except for the ones indicated with an asterisk (*) are our assumptions based on local expert knowledge. Consumer prices reflect the global trend. Please note that the prices of the products sold on direct markets in the SBL-Agri-SSP1 (milk, beef and cherries) are determined independently from the global trend. Labour requirement refers to labour hours required for each farm activity per hectare

Parameter category	Subcategory	Reference	SBL-Agri-SSP1	SBL-Agri-SSP2	SBL-Agri-SSP5	Source
Direct payments	Economic supports	100%	75%	100%	0%	Own assumption
	Ecological supports	100%	125%	110%	0%	Own assumption
Input prices	Hail insurance	100%	150%	150%	150%	Own assumption
	Fuel price	100%	*187%	*187%	100%	L.Felber & SFOE (2021)
Consumer prices	Food commodities	100%	*75%	100%	*88%	Doelman et al. (2018)
Efficiency	Labour requirement	100%	100%	90%	80%	Own assumption

#### SBL-Agri-SSP1: regional agriculture on the organic sustainable path

Society focuses on sustainable regional development with a high priority on small-scale, environmentally friendly production systems. Farm sizes slightly decrease, while the availability of farm workers increases. The share of large-scale livestock farms also decreases. Farming practices become substantially extensive: the use of mineral fertilisers and any kind of synthetic plant protection products are banned on both grassland and arable land. Cows are fed exclusively fresh grass and hay. The production of fodder, including legumes, on arable land is banned, except for cultivating clover grass to maintain soil nitrogen levels. Therefore, arable crops are cultivated only for human consumption, including soy, sunflower and grain maize, which are feasible due to climate change. Regional direct markets are well established, to the extent that they can offer high prices for locally produced farm products, such as cherries, milk and beef, whose prices are not influenced by the international market. The premium of the subsidies for environmental measures and EFAs (ecological focus areas: the area managed to promote farmland biodiversity) increases, whereas economic measures aimed at supporting agricultural production without considering environmental impacts, such as price support for cereals, are reduced. Furthermore, the conversion of grassland to arable land, currently possible, is banned.

#### SBL-Agri-SSP2: regional agriculture on the BAU (business-as-usual) path

Farm structures are gradually changing, following the path observed over the past 20 years (Helfenstein et al. 2022). As the average farm size slightly increases, farming efficiency also improves slightly due to increased livestock intensity and larger cattle barns. Larger livestock farms opt for a more efficient fodder system. Labour availability declines due to ongoing urbanisation. Most of the current regulations regarding the use of N fertilisers and synthetic chemical pesticides are maintained. However, as the current trend continues, EFA regulations will become more stringent. Also, a new biodiversity measure is introduced: 4% of arable land should be covered by EFAs. Climate change makes cultivating soy as fodder and sunflower and grain maize for human consumption an option. Livestock farms can intensify with mechanisation. The premium of the subsidies for environmental measures and EFAs increases slightly while remaining the same for economic measures.

#### SBL-Agri-SSP5: regional agriculture on the liberalised path

Society chooses to focus on economic efficiency, largely neglecting the provision and maintenance of ecosystem services. Thus, rapid structural change is expected. Only large, full-time farms can continue to operate, and part-time farms no longer exist. The field size doubles in comparison to the reference situation. With increasing dependence on fossil energy sources and other fossil-based inputs, the technical efficiency of farming, in terms of labour use, becomes the highest among the scenarios. Highly efficient large-scale livestock farms operate almost exclusively in this region, which contributes to the reduction of farm labour demand. The animals are housed in large cattle barns throughout the year and fed only hay and concentrate; pastures no longer exist. Grassland is used only for hay production. All subsidies, including those for extensively used orchard meadows, are eliminated. Instead, there are fewer limiting regulations. Similar to the SBL-Agri-SSP2, cultivation of soy as fodder, and sunflower and grain maize for human consumption are possible. The existing crop rotation rules, which can be flexible up to certain limits of the crop share on arable land, stay in place, given the assumption that farmers still attempt to maintain soil health. Only the percentage of grain corn acreage becomes less restrictive compared to the current legal restrictions on maximum crop shares.

### Integrated Land Use Change and Impact Assessment model

We employed the modelling framework developed by Nishizawa et al. (2022) to evaluate the impacts of the SBL-Agri-SSPs on agricultural land use. This framework had already been applied in the same study region. The present study extended it to capture the impacts of a wide array of agricultural land use drivers, as opposed to the cited study, which was limited to the evaluation of the current direct payment system. The result is “Integrated Land Use Change and Impact Assessment model (LUCIA),” a mathematical (linear) programming-based farm-level model that simulates the changes in agricultural land use for the defined scenarios at the farm level. The underlying assumption is that economically rational crop selections on individual farms lead to optimal land use, which in turn maximises the total gross margin (TGM) at the farm level. While maximizing TGM is only one possible driver for farmers’ behaviour, it aligns with the goals of a competitive farm seeking to optimize its chances of long-term financial stability (Hanley et al. 2012). The TGM is calculated by summing total revenues and subsidies, and then subtracting variable costs. This optimisation process was repeated for each of the farm types, typical for the study region (small dairy, large dairy, suckler, orchard and small farms), according to Nishizawa et al. (2022). The land use resulting at the farm level was aggregated across farm types, with weights based on farm size and the number of farms, to form the regional land use.

The farm activities in the farm-level model are constrained by four modules: land, input, feed and agricultural policy. The key assumptions of these modules are (1) no restrictions for converting grassland to arable land or vice versa, except for fields with a slope degree greater than 24%, which were defined as permanent grassland; (2) constant input use and livestock fodder requirements per unit of production; (3) maximum livestock capacity and (4) complete use of manure within a livestock farm. For the present study, new farm activities and management options were added to the existing model to be consistent with the scenario descriptions: new crops, an organic farming system without synthetic inputs, a grass-only fodder system and larger stable systems. We referred to De Ponti et al. (2012) and AGRIDEA (2020) for the yields of organically produced grass, crop and fruit (cherry). Farmers have the following options for the use of orchard meadows across scenarios: (1) turn them into commercial cherry production for regional direct markets (type A, 60 trees per hectare); (2) maintain existing orchards without harvesting cherries, thus only receiving subsidies (type B, 30 trees per hectare); (3) expand orchard meadows without cherry production (type C, 30 trees per hectare); (4) abandon the orchards. All meadows are extensively managed. The descriptions of all farm systems and structures across the developed scenarios can be found in Online Resource 2, along with the modelling parameters defined for each scenario. Online Resource 3 provides a complete list of the gross margins for all crop activities.

For the assessment of socioeconomic changes, we selected the following outputs: TGM (CHF ha^−1^ year^−1^), paid subsidy (CHF ha^−1^), TGM per labour hour (CHF h^−1^), N fertiliser use on farmland (kg ha^−1^), frequency of pesticide applications on arable land (times ha^−1^ year^−1^), livestock intensity on farmland (livestock units ha^−1^) and cereal, milk, beef and cherry production (kg per farm). The outputs for the ecological assessment are the number of trees, the area of EFAs and the biodiversity scores of individual species groups (ISGs) on farmland. The latter were obtained by coupling SALCA-Biodiversity (BD) (Jeanneret et al. 2014) with the farm model. In this model, each land use for arable land (crops with different intensities and flower strips) and grassland (meadow or pasture with different intensities), including orchards, received scores between 0 and 50 for eleven ISGs (arable land flora, grassland flora, birds, small mammals, amphibians, molluscs, spiders, carabids, butterflies, wild bees and grasshoppers). The scores were determined by the suitability of the land use for each ISG as well as the impacts of the chosen management options on the land. The average score of each land use was calculated based on the food web system on farmland. The biodiversity score at the farm level was calculated by aggregating the average scores of each land use into an area-weighted average. The regional biodiversity score was calculated by aggregating the biodiversity scores of each farm type with the same weights that were used for aggregating the farm-level results into the regional-level results. A detailed description of SALCA-BD can be found in Jeanneret et al. (2014) and Nishizawa et al. (2022).

### Data

We obtained the agricultural land use data for 2020 from the canton of Solothurn. These data include spatially explicit information on 4698 fields, containing the type of livestock, crops, management, the number of trees, area size and the average slope degree (ALW 2020). To determine reference grassland yields across intensities, we used a yield equation provided in GRUD (Agroscope 2017). Reference yields of arable crops were derived from regional yield data (2003–2020) for Canton Solothurn (Erdin 2021). We assumed that these yields corresponded to the yield level for extensive management recorded in AGRIDEA because the region’s predominant farming system reduces pesticide inputs. We referred to AGRIDEA (2020) for the yield levels for intensive and organic management as well as the gross margins of crops and livestock.

### Validation of the scenarios and parametrisation

To validate the SBL-Agri-SSP narratives, we ensured both horizontal and vertical consistency, as suggested by Mitter et al. (2020). The horizontal consistency was checked by assessing the internal consistency across different scenario components within each scenario and across different scenarios, while the vertical consistency was ensured by assessing the consistency across different spatial scales (with the Eur-Agri-SSPs). The initial parametrisation for the reference scenario was validated in the following ways: first, we ran the farm-level model to retrieve the reference land use, which parameters were based on the current data in 2020. Second, we compared the reference land use with the observed land use. In the case of a deviation, we examined the parametrisation of farm activities and constraints and reran the model until the deviation was minimised. This process was repeated for all modelled farm types. Some deviations from the current observation were allowed to realistically model farm activities. For example, the assumption of a specific number of trees per hectare led to a deviation of the total number of trees simulated for the region. This was deemed necessary to maintain a realistic model of orchard meadows as the number of trees within any given field may vary considerably. Furthermore, we verified whether the results simulated by the future scenarios were within plausible ranges by referring to the observation and historical land use data.

## Results

### Major agricultural land use changes

**Fig. 3:**
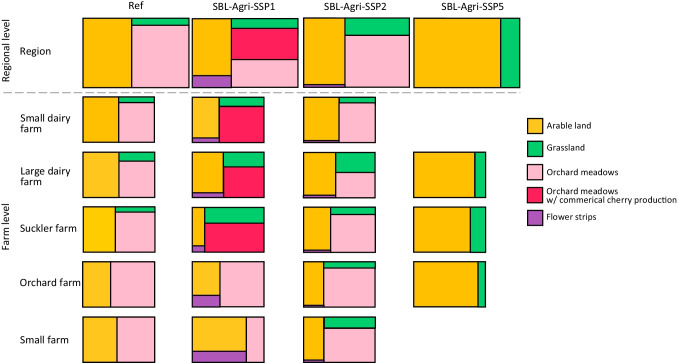
Agricultural land uses across the reference and the SBL-Agri-SSP scenarios at the regional level and farm level. Ref is the reference scenario

Figure 3 presents the agricultural land uses, including grassland, orchard meadows with and without commercial cherry production, arable land and flower strips, across the scenarios at the regional and farm levels, simulated with LUCIA given the framework conditions of SBL-Agri-SSPs. Table 3 shows the shares of the regional agricultural land in terms of farming management, fodder production and new crop adoption due to climate change. The reference (Ref) is the modelling result with the input data for 2020. In the SBL-Agri-SSP1, a distinctive change is observed in orchard meadows and flower strips: more than half of the orchard meadows are now used for commercial cherry production, whereas they were previously used exclusively for fodder production, without harvesting cherries. Flower strips as a measure of EFA on arable land, account for around 15% of the arable land. However, for smaller-scale farms (i.e. orchard and small farms), commercial cherry production is still too costly to be an option. The use of grassland increases, but the management intensifies (more cuts per year) due to the roughage-only fodder system, which is demanded under this scenario. In the SBL-Agri-SSP2, the land use share remains similar to the reference scenario. However, more arable land is under extensive management due to higher premiums for ecological measures. Grassland is managed slightly more intensively, similar to the SBL-Agri-SSP1. This is due to the assumed higher livestock capacity on large dairy farms. Consequently, the area of orchard meadows on these farms declines. In the SBL-Agri-SSP2, orchard meadows continue to be used only for conservation purposes: the trees are maintained to receive subsidies. Compared to the regional land use in the reference scenario, a considerable change is observed in the SBL-Agri-SSP5: orchard meadows completely disappear, while grassland is minimised only in the areas with steep slopes and becomes intensively managed. Consequently, most of the farmland is utilised for arable crop cultivation with intensive management. Because of more efficient fodder production, more arable land is allocated for cereal production. The new crops that are likely to be adopted in the case study region are profitable enough to be chosen in LUCIA in all scenarios.

**Table 3:** Shares of the regional agricultural land in terms of farming management, fodder production and the adoption of new crops across the reference and SBL-Agri-SSP scenarios. The grassland with orchards is assumed to be extensively managed. The share of new crops indicates the share of the area utilised for the new crops on the whole arable land in the region. Ref is the reference scenario

Regional land use	Management	Ref	SBL-Agri-SSP1	SBL-Agri-SSP2	SBL-Agri-SSP5
Grassland (100%)	Intensive*	11%	26%	20%	100%
	Extensive	89%	74%	80%	0%
Arable land (100%)	Intensive	70%	0%	44%	86%
	Extensive	30%	0%	52%	14%
	Organic	0%	100%	0%	0%
Fodder production area		72%	69%	70%	40%
Cereal production area		28%	31%	30%	60%
Share of new crops (within arable land)	Soy	-	13%	0%	0%
	Grain maize	-	40%	5%	23%
	Sunflower	-	0%	25%	0%

*Intensity of grassland is determined by the number of cuts

### Trade-offs of agricultural land use changes

Table 4 presents the relative changes of the key socioeconomic and ecological indicators across scenarios and farm types in comparison to the reference scenario. The numerical results of all the examined indicators can be found in Online Resource 4 (Table S4.2).

**Table 4:**
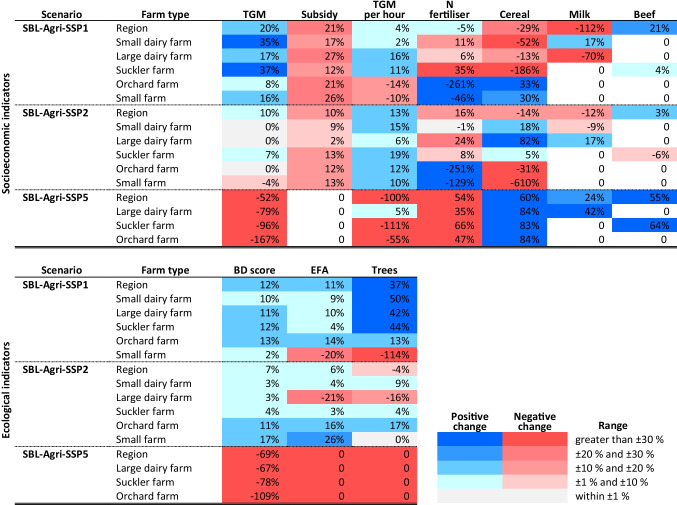
Relative changes of the resulting key socioeconomic and ecological indicators calculated across the SBL-Agri-SSP scenarios simulated with LUCIA at the regional level and across farm types in comparison to the reference scenario. *TGM* total gross margin, *EFA* ecological focused area. Positive and negative increases are shown in shades of blue and red, respectively

#### Regional level

In the SBL-Agri-SSP1, despite the largest decline in food prices (Table 2), the total gross margin (TGM) increases the most among all scenarios (+ 20%). The biodiversity score also increases the most (+ 12%). However, an increase in subsidy payments is also the highest (+ 21%), despite the reduction in income support. Against this highest TGM growth, the TGM per required labour hour (hereafter TGM per hour) shows only a modest rise (+ 4%), compared to that in the SBL-Agri-SSP2 (+ 13%), indicating more labour-intensive farming in this scenario. In terms of farm production levels in SBL-Agri-SSP1, beef production increases (+ 21%) due to the assumed increase in the number of sucker farms, and the number of trees also substantially increases (+ 37%) due to the introduction of commercial cherry production. However, both cereal and milk production decline (− 29% and − 112%). In the SBL-Agri-SSP2, the TGM increases modestly (+ 10%) and the biodiversity score as well (+ 7%) paralleling an increase in EFAs (6%). However, the N fertilisers use increases (+ 16%). In the SBL-Agri-SSP5, both the TGM and the TGM per hour fall considerably (− 52% and − 100%). This effect was found despite an increase in farm production in all categories except for cherry, as shown by an increase in cereals (+ 60%), milk (+ 24%) and beef (+ 55%). These higher production levels were achieved through larger livestock capacities and the specialisation of crops for fodder production, which mainly relies on concentrates. The intensification led to higher use of N fertilisers (+ 54%) and a decline in the biodiversity score (− 69%), followed by the complete abandonment of EFAs and orchard trees.

#### Farm level

The TGM growth for livestock farms is particularly strong in the SBL-Agri-SSP1. However, large dairy farms produce much less milk (− 70%) due to the reduced livestock capacity, while small dairy farms produce more (+ 17%). The revenue loss from the sale of milk is offset by the assumed higher milk price (0.6 CHF kg^−1^ to 0.9 CHF kg^−1^). In the SBL-Agri-SSP2, while the biodiversity score of large dairy farms increases (+ 3%), this farm type uses significantly more N fertilisers and reduces the area of EFAs and the number of trees. In the SBL-Agri-SSP5, all farm types experienced an extensive loss of TGM given the assumed larger farm size and the elimination of subsidies, but these declines are more distinct for suckler and orchard farms (− 96% and − 167%), which rely on subsidies for their income more than large dairy farms do in the reference scenario.

**Fig. 4:**
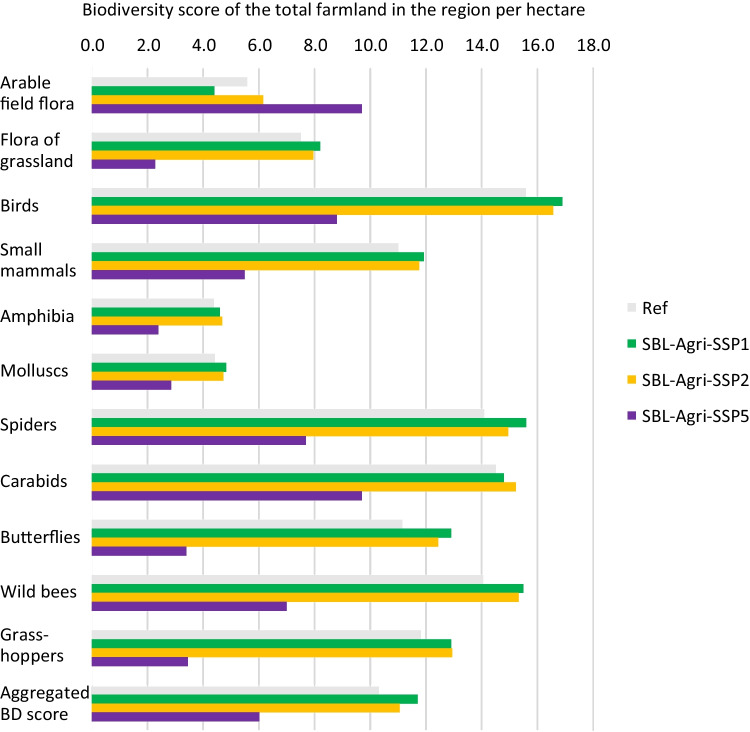
Absolute change in the biodiversity (BD) scores of individual species groups and the aggregated scores over species groups. The biodiversity scores are area-weighted averages for the whole regional farmland

Figure 4 depicts the differences in biodiversity scores across ISGs, indicating the extent to which agricultural land use potentially impacts them. Even though the overall score of the SBL-Agri-SSP5 decreases considerably (− 41%), the score for the arable field flora is higher than the reference. This particular score decreases in the SBL-Agri-SSP1, while all other biodiversity scores increase, reflecting the change in the shares of grassland and arable land. These land use changes also translate to the scores of fauna species that particularly depend on (species rich) grassland. The scores of butterflies, wild bees and grasshoppers are reduced by more than 50% in the SBL-Agri-SSP5 as compared to the other scenarios. The scores for carabid beetles, which are also related to arable land, and for farmland, birds were also significantly reduced as both are impacted by the disappearance of fruit orchards in the SBL-Agri-SSP5.

## Discussion

### Implications for promoting diversified agricultural landscapes

Contrasting land use changes were observed regarding agricultural diversification across the investigated scenarios. In the SBL-Agri-SSP1 (regional agriculture on the organic sustainable path), commercial cherry production became profitable, and the total number of trees increased substantially, reaching 1981 levels (BfS 1983, the last tree census), and a large area of arable land was also converted into flower strips. This result is primarily due to an assumed higher premium for extensive grassland and other ecological measures and a higher cherry price (1.2 CHF kg^−1^ to 3.8 CHF kg^−1^), driven by consumer preferences, i.e. higher demand for local produce. These assumptions mitigated the negative impact of the decline in food commodities. Our sensitivity analysis on cherry prices showed that 3.7 CHF kg^−1^ is the threshold for commercial cherry production to be sufficiently incentivised. Consequently, the highest level of farmland biodiversity was achieved in this scenario. The SBL-Agri-SSP2 (regional agriculture on the BAU path) maintains the current level of biodiversity, but uses N fertilisers more extensively. This presents a challenge to the core environmental objectives in the Swiss agricultural policy from 2022 and 2050 (Schweizer Bauernverband 2020; Schweizerischer Bundesrat 2022). Without financial support, orchard trees are in danger of abandonment (Mack 2017). The SBL-Agri-SSP5 (regional agriculture on the liberalised path) demonstrates that the potential for multifunctional use of agricultural land comes at the expense of an increased supply of farm products (i.e. specialising in intensive crop production and abandoning orchard meadows). Agricultural land use in crop monocultures could lead to a decline in soil functionality and productivity (Gregorich et al. 2011), and potentially increase the risk of infectious diseases and pest outbreaks (Keesing et al. 2010; Civitello et al. 2015), apart from the degradation of biodiversity (Marques et al. 2019; Raven & Wagner 2021).

In addition to prices and subsidies, various socioeconomically related assumptions such as the choice of fodder systems, livestock intensity and labour supply affected the multifunctional nature of regional agriculture and biodiversity. As shown in the SBL-Agri-SSP2, large dairy farms with the same fodder system as the reference scenario but with higher livestock capacity led to a reduction in the overall EFAs on the farms. The loss of biodiversity on grassland was, however, compensated by the mandatory implementation of EFAs on arable land. Regarding labour supply, in the SBL-Agri-SSP1, securing sufficient farm labour is a prerequisite for the agricultural system assumed in this scenario, which is highly labour-intensive due to the total ban on synthetic inputs, exclusive grass-based fodder systems and commercial cherry production. This scenario assumed a 14% increase in regional farm labour compared to the reference scenario, which could pose a legitimate challenge for this scenario, given a general trend of declining farm populations in advanced countries (Eurostat 2022). In the SBL-Agri-SSP5, higher agricultural efficiency was considered, which reduced the labour demand for field activities by 20% and for managing dairy cows by 30%. Nonetheless, large dairy farms could not reach the maximum livestock capacity due to the lower availability of labour. The sensitivity analysis showed that one additional full-time labour unit (2600 h) is required to generate almost the same TGM as obtained in the reference scenario.

Compared to the impacts of these changed socioeconomic conditions, the long-term impacts of climate change through yield changes were minor in this study (Online Resource 1). This is not only because the estimated yield changes from the baseline remained rather marginal, independent of the chosen climate scenario, but also because agriculture in Switzerland is highly dependent on the subsidy system. This finding supports the claim that agricultural land use in Switzerland is less sensitive to climate change than other drivers (Lehmann et al. 2013; Klein et al. 2014; Fronzek et al. 2019). Instead, in all scenarios, the more noticeable impacts of climate change appeared as the adaptation of new crops, as the farmers’ choice of the new crops in the model generated higher incomes.

The characteristics of farms play an important role in influencing changes in agricultural land use (van Vliet et al. 2015) and the effectiveness of agricultural policies (Huber et al. 2023). By accounting for farm heterogeneity, we also demonstrated differences in the sensitivity of farm-level indicators among farm types to specific drivers. Because suckler and orchard farms rely more on subsidies than dairy farms, their TGMs are sensitive to changes in the subsidy scheme. Particularly in cases in which subsidies are eliminated, the economic losses for these farm types could be substantial. Differences were also observed in the adoption of EFAs. There was no incentive for livestock farms to implement EFAs on arable land beyond the minimum required. In the SBL-Agri-SSP2, large dairy farms even reduced EFAs and orchard meadows, despite an increase in the premium for ecological measures. Additionally, our assumption that smaller farms face significant structural changes, e.g. no more small and part-time farms in the SBL-Agri-SSP5 aligns with Arnalte-Mur et al. (2020) who found that small farms are highly impacted by social changes.

This study uncovered several important trade-offs. The increase in subsidy expenditure was unavoidable to promote biodiversity and diverse agricultural landscapes, driven by commercial cherry production and more ecological measures. The increase in the TGM for farms does not necessarily imply a commensurate increment in the rate of TGM per hour. Another trade-off appeared between production level and farmland biodiversity. The extensification of agricultural practices in the SBL-Agri-SSP1 scenario increased the biodiversity level but resulted in a substantial reduction in cereal and milk production. Conversely, the agricultural intensification in the SBL-Agri-SSP5 scenario led to a significant increase in all farm produce, excluding cherries, which came at the expense of biodiversity. This finding confirms many other previous studies that demonstrated the trade-off between intensification and specialisation in agricultural systems and biodiversity (Klasen et al. 2016; Dudley & Alexander 2017; Beckmann et al. 2019; Zabel et al. 2019), implying that increasingly stringent agri-environmental regulations may be linked to national food security issues. Mann and Kaiser (2023) found that the failure of recent ambitious agri-environmental objectives in Switzerland stems from insufficient measures to maintain a national self-sufficiency rate, while Finger and Möhring (2022) argued that the implementation of synthetic pesticide-free production in Switzerland necessitates a diverse array of policy instruments that extend beyond purely financial incentives. Correspondingly, this study recommends that food and agri-environmental policies should address broader issues that promote diversified agriculture while acknowledging diverse impacts across different farm types and potential challenges and trade-offs. These encompass securing a rural agricultural workforce to mitigate declining farm populations (see Dutta et al. 2017), maintaining national food self-sufficiency, managing food consumption preferences and patterns that potentially contribute to an increase in prices of locally produced products (see Mann & Kaiser 2023) and implementing feed systems and livestock intensities that inhibit the extensive use of arable land.

### Limitations of the study

This study’s integrated model-based scenario approach addressed scale issues related to investigating the impacts of global future pathways on regional agricultural land use. The approach enables comparative studies in other Swiss and European regions under the common SSP scenario framework, considering the regional variation in the implications of global drivers (Vanbergen et al. 2020; Debonne et al. 2022). This study, however, did not implement an even smaller reference unit (i.e. parcel or field) for decision-making that could connect with real physical entities as opposed to the more abstract entities that were considered. Modelling at a high spatial resolution can generate more refined future projections based on individual farmers’ decisions and then facilitate stakeholder engagement in scenario development (Brown & Castellazzi 2014). We also identified three major limitations to our applied farm-level model. First, even though our focus was on comparative analysis across scenarios, the static nature of our farm-level model could not account for the dynamic process of farm management and development over time. For example, substantial investments that require long-term decisions might be needed to realise the structural changes to farms and the improvement of productivity assumed in the scenarios (Neuenfeldt et al. 2019; Giller et al. 2021). Orchard planning also happens on a decade-by-decade basis rather than yearly. Second, in the interviews conducted by Suškevičs et al. (submitted), the stakeholders in the same study region mentioned various agricultural technologies that could potentially be adopted. However, our ability to explicitly consider the impacts of technologies was limited. These impacts were reflected only in the assumed changes in prices and labour requirements. Lastly, even though data on farm labour use and the agricultural workforce are often remarkably inaccurate (Nye 2018), it is crucial to consider a flexible farm labour supply, especially when structural changes in a farm reduce family farm labour, as anticipated in the SBL-Agri-SSP5. The change in agricultural efficiency through investment in agricultural technology is a key determinant of agricultural land use dynamics (Popp et al. 2017; Stehfest et al. 2019). Future studies should explicitly consider the adoption of new agricultural technologies and explore how the associated efficiency changes would impact farm and seasonal labour demand as well as agricultural land use patterns. Eventually, other climate change effects could have a considerable influence on yield patterns. For example, CO_2_ fertilisation effects might at least partially compensate for potential reductions in plant growth and net primary production (Leung et al. 2022). Nonetheless, it seems exceedingly optimistic to posit that the effects of increased ambient CO_2_ may completely manifest as yield improvements (Long et al. 2004, 2006; Wang et al. 2020). Furthermore, given the anticipated increase in drought frequency and severity in large parts of Europe, including Switzerland (Grillakis 2019; Vicente-Serrano et al. 2022), one should carefully interpret our results concerning climate change within the limitations of the approach we employed for the incorporation of climate change into LUCIA.

## Conclusions

This study identified the socioeconomic implications of agricultural LUCs for promoting agriculturally diverse land uses in the long term. By explicitly accounting for farm heterogeneity and regional characteristics of socioeconomic and climatic conditions, we addressed the scale issues that inevitably arise when examining the effects of global drivers on regional agricultural land use impacted by farm-level decisions. To our knowledge, this study is the first to downscale the Eur-Agri-SSPs to a small region representing multifunctional agricultural landscapes in Switzerland and infer regional future scenarios. The results suggest that food and agri-environmental policies need to consider a broader range of land use drivers beyond financial support for farms for future agricultural diversification while acknowledging potential trade-offs and diverse impacts across different farm types. We conclude that our established approach, which simulated agricultural land use changes on a smaller scale with various socioeconomic and climate conditions that reflect regional trends and traits, is easily applicable to other regions in Europe and allows us to envision a wider set of tangible future implications across these regions towards desired pathways.

### Supplementary information

Below is the link to the electronic supplementary material.

Supplementary file1 (DOCX 932 KB)

Supplementary file2 (DOCX 39 KB)

Supplementary file3 (DOCX 32 KB)

Supplementary file4 (DOCX 40 KB)

## Data Availability

The datasets analyzed during the current study are available from the corresponding author on request.
