# Supplementary material for: Towards diverse agricultural land uses: socio-ecological implications of European agricultural pathways for a Swiss orchard region
Source: Reg Environ Change. 2023 Jul 22;23(3):97. doi: 10.1007/s10113-023-02092-5 (PMC10363045; doi:10.1007/s10113-023-02092-5)
Supplement: Supplementary file 1 — Supplementary file1 (DOCX 932 KB) [file 10113_2023_2092_MOESM1_ESM.docx]

*Regional Environmental Change*

**Online Resource 1 (Climate impact assessment)**

Towards diverse agricultural land uses: socio-ecological implications of European agricultural pathways for a Swiss orchard region

Takamasa Nishizawa^*^, Sonja Kay, Johannes Schuler, Noëlle Klein, Tobias Conradt, Michael Mielewczik, Peter Zander, Joachim Aurbacher, Felix Herzog

*Corresponding author: Takamasa Nishizawa, Leibniz Centre for Agricultural Landscape Research (ZALF) e.V., Farm Economics and Ecosystem Services, Müncheberg, Germany

E-Mail: [takamasa.nishizawa@zalf.de](mailto:takamasa.nishizawa@zalf.de); Tel.: +49 (0)33432 82-490; Fax: +49 (0)33432 82-4082

# Projected climate change

Climate change under the RCP 4.5 and 8.5 emission scenarios was represented by CMIP5-based EURO-CORDEX simulations with a spatial resolution of approximately 12.5 km (Coppola et al., 2021; Jacob et al., 2014). These contain daily realizations of meteorological parameters (air temperature, precipitation, solar radiation, wind speed etc.) for the years 2006–2099 from different global and regional weather model combinations (model chains). Two to three dozen of such realisations per scenario had been bias-adjusted using the so-called ISIMIP-2b approach described by Hempel et al., (2013). In addition, we could access 31 years of Meteoswiss climate station observations from the two Swiss cantons Solothurn and Basel-Landschaft located in and around the study region, and we made use of the 0.1-degree E-OBS data version 24.0e. (Cornes et al., 2018). Fig.S1.1 shows the resulting climate scenarios until 2100 for the regions.


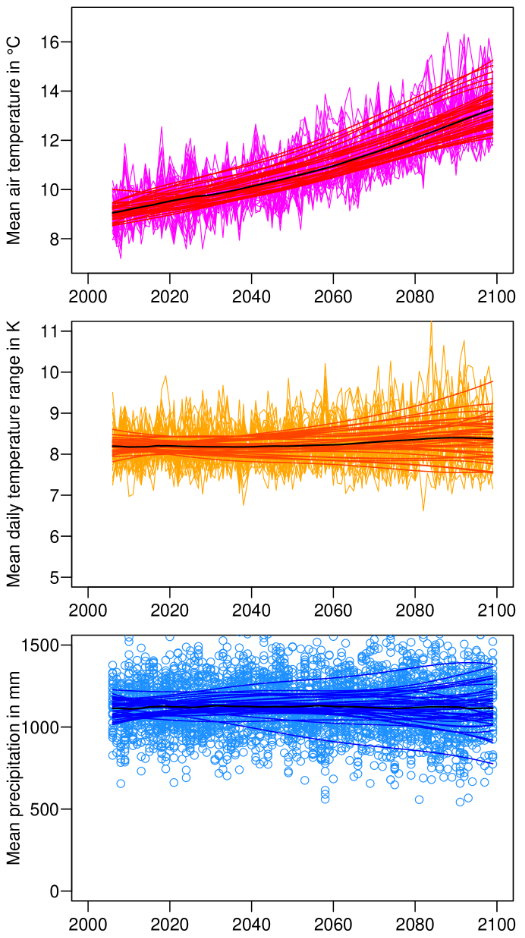


**FigS1.**1 Climate scenarios for the two Swiss cantons (Solothurn and Basel-Landschaft). The black lines are the medians of the model trajectories (coloured lines) smoothed from their annual averages. Future crop yield prediction

# Future crop yield prediction

We used estimated yield changes of the most important crop species relative to their current yield levels as model input into LUCIA, together with the SBL-Agri-SSPs. The crop yield estimations were generated with the model ABSOLUT (Conradt, 2022) which uses observed weather and yield data to determine the climate sensitivity profiles for different crops in regional discretisation. Based on this setup and calibration, crop yield scenarios can be calculated from climate scenario data.

The yield model was applied to a larger area consisting of Switzerland, Austria, and the Southern-German federal states Baden-Wurttemberg and Bavaria, the spatial discretisation were cantons in Switzerland, districts in Germany, and 25 km grid cells in Austria. Yield data for numerous crops was obtained through national authorities in this spatial discretisation. Historical weather data were extracted from E-OBS v.24.0e. To estimate future crop yields we used the bias-adjusted EURO-CORDEX climate scenarios detailed above.

The ABSOLUT model builds on spatially distributed multivariate regressions whose pre-harvest weather parameters are automatically selected based on past observations of weather and crop-specific yields. Spatial cross-validation and strict out-of-sample handling of training data allow for reliable predictive performances regarding the pure weather/climate change effect; CO₂-fertilization or changes in management are not represented.

Fig. S1.2 presents the expected relative crop yield changes in 2050 compared to the 2020 yield levels estimated with ABSOLUT. The data for each reference year are aggregated from 21 years of model output (e.g. 2040–2060 for 2050). The width of each bar exhibits the standard deviation of inter-annual yield change, which indicates a crop-specific yield risk. An additional variation in the original yield projections caused by the different climate realisations was eliminated by using the median yield level of all realizations. The inter-annual variance was also determined as the median variance from all realizations. To compare the magnitude of crop risks over crops, it was normalised by the median of each crop. Nonetheless, we excluded the crop risks as model input for LUCIA: clear trends regarding the crop risks could not be detected between 2020 and 2050, RCP4.5 and RCP8.5.


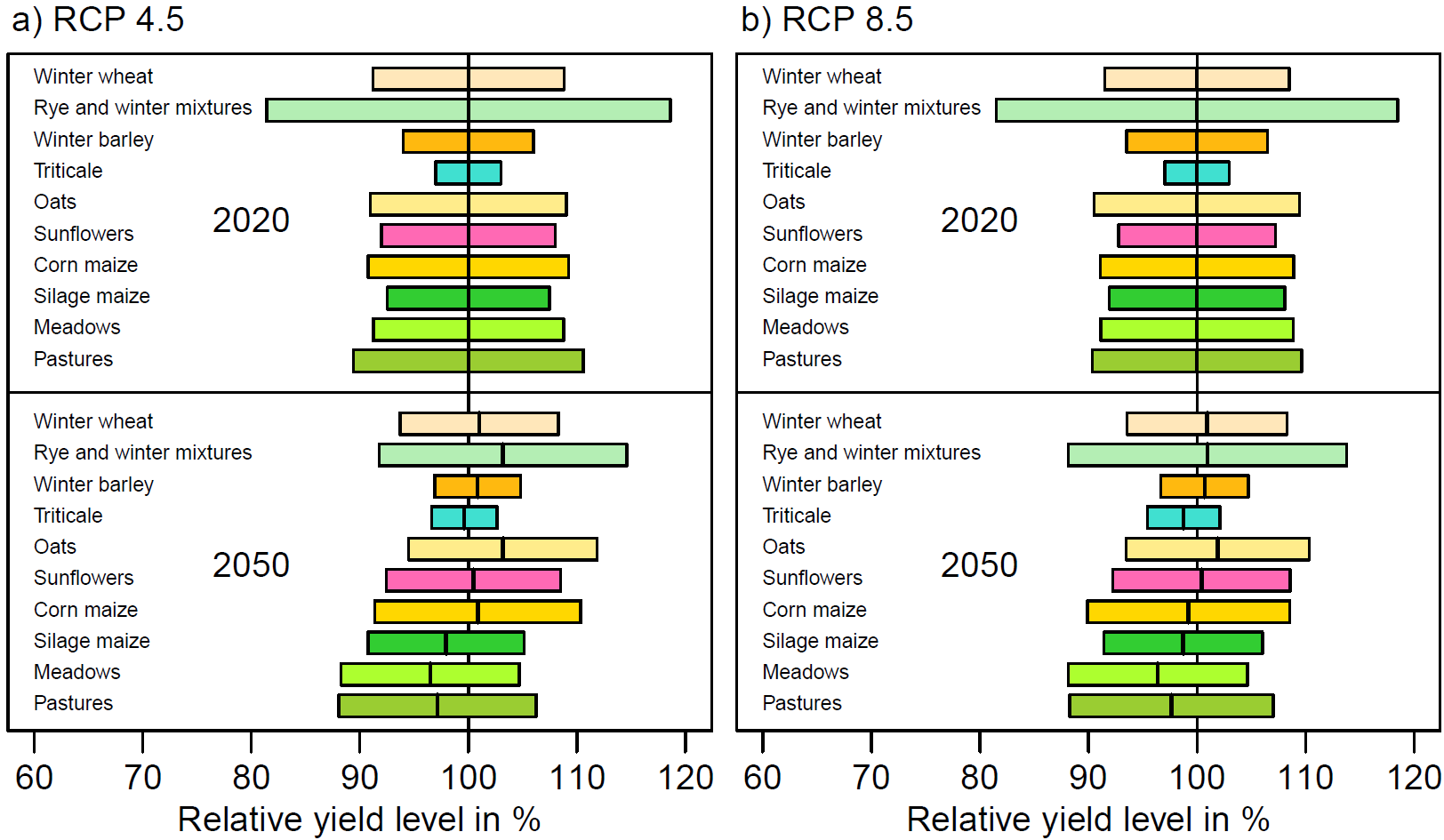


**Fig S1.2** Crop yields in 2050 relative to 2020 depending on RCP 4.5 and 8.5 projected with the ABSOLUT model in box plots. The width of the bars indicates the standard deviation normalised by the median of the interannual variation in the relative yield level, and the vertical line in the bars indicates the relative yield change implemented into the LUCIA

# Climate analogue regions

Along with the prediction of crop yields in the study region, this study considered a new cropping system for LUICA that has not yet been adopted by the farmers in the study region but is likely to be adopted in 2050. This was supported by identifying so-called climate analogue regions, following Mahony et al. (2017). The basic idea of climate analogue regions is, given a projection of the future climate of a certain location P, to identify another location or region Q that is currently experiencing a climate that matches or is at least close to what is expected for P. Then it is informative to look for the differences between the crop portfolios actually grown at P and Q. Respective maps of climate similarity for the years 2050 and 2080 were calculated in the EURO-CORDEX grid based on present and future average seasonal temperatures and precipitation depths (2 ⨉ 8 variables per calculation/map, cf. Mahony et al., 2017) using the raster cells covering the cantons Basel-Landschaft and Solothurn as starting point. In addition, we referred to the Soil Atlas of Europe (Jones et al. 2005) and the European Soil Database (ESDAC 2004) to consider environmental differences. Crop area statistics in NUTS-2 regions collected by Eurostat were used to determine shifts in crop species between the study region and its climate analogues.

Fig.S1.3 displays the analogue regions identified by the climate scenario data. The current climate in the areas indicated by light blue tints is expected to be similar to the study region’s climate in 2050. Besides a larger area in Eastern France, they stretch across the two cantons themselves (Solothurn and Basel-Landschaft) plus some adjoining regions such as the Aare valley with parts of the cantons Aargau and Luzern. Therefore, we selected the most likely new cropping systems in 2050 for our case study region by referring to the typical crops cultivated in these regions: they are soy, sunflowers and grain maize. They were added to a list of the crops in LUCIA.


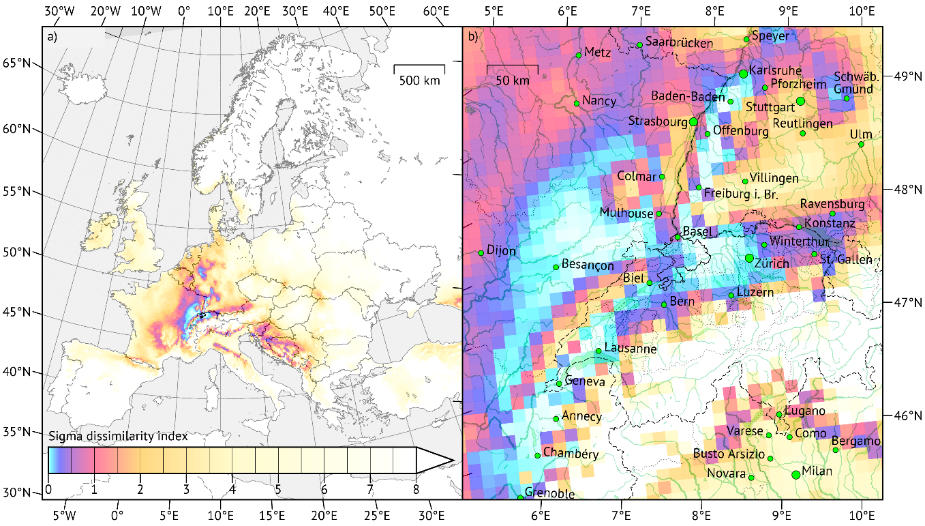


**Fig. S1.3** The 2050 climate analogue index map for the two Swiss cantons (Solothurn and Basel-Landschaft)

# References

Conradt, T. (2022). Choosing multiple linear regressions for weather-based crop yield prediction with ABSOLUT v1.2 applied to the districts of Germany. *International Journal of Biometeorology*, *1*, 3. https://doi.org/10.1007/s00484-022-02356-5

Coppola, E., Nogherotto, R., Ciarlo’, J. M., Giorgi, F., van Meijgaard, E., Kadygrov, N., Iles, C., Corre, L., Sandstad, M., Somot, S., Nabat, P., Vautard, R., Levavasseur, G., Schwingshackl, C., Sillmann, J., Kjellström, E., Nikulin, G., Aalbers, E., Lenderink, G., … Wulfmeyer, V. (2021). Assessment of the European Climate Projections as Simulated by the Large EURO-CORDEX Regional and Global Climate Model Ensemble. *Journal of Geophysical Research: Atmospheres*, *126*(4), e2019JD032356. https://doi.org/10.1029/2019JD032356

Cornes, R. C., van der Schrier, G., van den Besselaar, E. J. M., & Jones, P. D. (2018). An Ensemble Version of the E-OBS Temperature and Precipitation Data Sets. *Journal of Geophysical Research: Atmospheres*, *123*(17), 9391–9409. https://doi.org/10.1029/2017JD028200

Hempel, S., Frieler, K., Warszawski, L., Schewe, J., & Piontek, F. (2013). A trend-preserving bias correction &ndash; The ISI-MIP approach. *Earth System Dynamics*, *4*(2), 219–236. https://doi.org/10.5194/ESD-4-219-2013

Jacob, D., Petersen, J., Eggert, B., Alias, A., Christensen, O. B., Bouwer, L. M., Braun, A., Colette, A., Déqué, M., Georgievski, G., Georgopoulou, E., Gobiet, A., Menut, L., Nikulin, G., Haensler, A., Hempelmann, N., Jones, C., Keuler, K., Kovats, S., … Yiou, P. (2014). EURO-CORDEX: New high-resolution climate change projections for European impact research. *Regional Environmental Change*, *14*(2), 563–578. https://doi.org/10.1007/S10113-013-0499-2/FIGURES/8
