# Supplementary material for: Towards diverse agricultural land uses: socio-ecological implications of European agricultural pathways for a Swiss orchard region
Source: Reg Environ Change. 2023 Jul 22;23(3):97. doi: 10.1007/s10113-023-02092-5 (PMC10363045; doi:10.1007/s10113-023-02092-5)
Supplement: Supplementary file 2 — Supplementary file2 (DOCX 39 KB) [file 10113_2023_2092_MOESM2_ESM.docx]

*Regional Environmental Change*

**Online Resource 2 (Scenario description and parameter setting)**

Towards diverse agricultural land uses: socio-ecological implications of European agricultural pathways for a Swiss orchard region

Takamasa Nishizawa^*^, Sonja Kay, Johannes Schuler, Noëlle Klein, Tobias Conradt, Michael Mielewczik, Peter Zander, Joachim Aurbacher, Felix Herzog

*Corresponding author: Takamasa Nishizawa, Leibniz Centre for Agricultural Landscape Research (ZALF) e.V., Farm Economics and Ecosystem Services, Müncheberg, Germany

E-Mail: [takamasa.nishizawa@zalf.de](mailto:takamasa.nishizawa@zalf.de); Tel.: +49 (0)33432 82-490; Fax: +49 (0)33432 82-4082

**Table S2.1** Detailed scenario description. All the assumptions are intended to be consistent with the storylines of the corresponding Eur-Agri-SSPs (Mitter et al., 2020).

| **Scenario element** | **Sub-element** | **SBL-Agri-SSP1 (Regional agriculture on the organic sustainable path)** | **SBL-Agri-SSP2 (Regional agriculture on the BAU path)** | **SBL-Agri-SSP5 (Regional agriculture on the liberalisation path)** |
| --- | --- | --- | --- | --- |
| **Population and urbanisation** | Population growth | Low | Medium, due to the spatial proximity to Basel | High, more people commute to Basel |
|  | Urbanisation speed | Medium, while more people live in rural areas | Medium | High, no more current restrictions on building new houses |
|  | Environmental awareness of citizens | Higher | Same, more protection of rural landscapes | Lower |
|  | Average age of farming population | Younger | Same as the reference | Younger |
|  | Average educational level of the farmers | Higher, more innovative farmers and efficient farming | Same as the reference | Higher |
|  |  |  |  |  |
| **Economy and markets** | Prices (fuel, labour) | Increase | Increase | Increase but fuel price remains the same |
|  | Prices (crops, feed, livestock products) | Decrease, while higher price for direct markets | Same as the reference | Slightly decrease |
|  | Connectivity to the global market | Lower | Same as the reference | Higher |
|  | Direct markets | Well developed, as higher demand for locally grown food | Same as the reference | Nearly dissolved |
|  | Demand for feed | Lower, only-grass-based feed | Same as the reference | Higher demand for concentrates |
|  |  |  |  |  |
| **Policies and institutions** | Economic support for farmers | Lower, less support for intensive management | Same as the reference | None |
|  | Payments to environmental measures | Higher, more support for extensification | Slightly increase | None |
|  | Conservation standards | Higher | Medium | Strongly reduced |
|  | Biodiversity measures | Higher, improve connectivity of wildlife habitats | Medium, slightly higher financial support | No financial support |
|  |  |  |  |  |
|  |  |  |  |  |
|  |  |  |  |  |
|  |  |  |  |  |
|  |  |  |  |  |
| **Technology** | Adoption rate of new technology | High, especially biological and environmentally friendly machines | Moderate technological advances | Strongly high, especially for reducing labour input |
|  | Automatization | Medium | Medium | Strongly high |
|  | Possible technologies | Controlled traffic farming for spotted wing drosophila |  | Irrigation, precision farming |
|  |  |  |  |  |
| **Environment and natural resources** | Green infrastructure | Higher | Medium, preservation of orchards trees, biodiversity measures on arable land | Strongly reduced |
|  | Renewable energies | Higher, such as solar, wind and water | Medium | Mostly with fossils |
|  |  |  |  |  |
| **Farm structure** | Structural change | Not distinctively happen | Slowly happen | Rapidly happen |
|  | Number of farms | Slightly increase (77 farms in the region)  ---------------------------------------  Orchard farm (11 farms)  Small dairy farm (10 farms)  Large dairy farm (8 farms)  Suckler farm (21 farms)  Small farm (27 farms) | Decrease (67 farms in the region)  ---------------------------------------  Orchard farm (6 farms)  Small dairy farm (8 farms)  Large dairy farm (11 farms)  Suckler farm (19 farms)  Small farms (23 farms) | Strongly decrease (30 farms in the region)  ---------------------------------------  Orchard farm (3 farms)  Small dairy farm (0 farms)  Large dairy farm (13 farms)  Sucker farm (14 farms)  Small farm (0 farms) |
|  | Farm size | Slightly smaller (23 ha: regional average)  ---------------------------------------  Orchard farm (40 ha)  Small dairy farm (20 ha)  Large dairy farm (35 ha)  Suckler farm (30 ha)  Small farm (8.5 ha) | Larger (26 ha: regional average)  ---------------------------------------  Orchard farm (42 ha)  Small dairy farm (22 ha)  Large dairy farm (45 ha)  Suckler farm (35 ha)  Small farm (8.5 ha) | Strongly larger (59 ha. regional average)  ---------------------------------------  Orchard farm (35 ha)  Large dairy farm (75 ha)  Suckler farm (50 ha) |
|  | Farm labour availability  (1 AWU = 2600 hours) | Higher (+14% in the region)  --------------------------------------  Orchard farm (1.0 AWU)  Small dairy farm (1.5 AWU)  Large dairy farm (1.6 AWU)  Suckler farm (1.5 AWU)  Small farm (0.4 AWU) | Lower (-20% in the region)  ---------------------------------------  Orchard farm (0.3 AWU)  Small dairy farm (1.0 AWU)  Large dairy farm (1.7 AWU)  Suckler farm (1.2 AWU)  Small farm (0.2 AWU) | Strongly lower (-50% in the region)  ---------------------------------------  Orchard farm (0.2 AWU)  Large dairy farm (1.6 AWU)  Suckler farm (1.0 AWU) |
|  | Full-time farms | Decrease | Slightly decrease | Increase |
|  | Part-time farms | Increase | Slightly increase | Not exist |
|  |  |  |  |  |
| **Farming systems** | Field size | Smaller | Same (0.7ha) (10% less labour requirements) | Larger (20% less labour requirements) |
|  | Mineral fertilisers | Not allowed | Allowed | Allowed |
|  | Plant protections | Not allowed | Allowed | Allowed |
|  | Organic | Modelled (Cherry yield loss –22%, Grass yield loss –11% (De Ponti et al., 2012), yield of organic arable crops based on AGRIDEA) | Not modelled | Not modelled |
|  | Crop rotation | Same as SBL-Agri-SSP2 and nitrogen fixation (area of Ley/soy and other crops must be 1 : 2) | Cereals (without maize/oats) < 66%  Wheat/spelt/triticale < 50%  Oats < 25%  Maize < 40%  White peas < 15% | Same as ABL-Agri-SSP2 except for maize < 75% |
|  | New cropping systems | Corn maize (organic)  Sunflower (organic)  Soy (organic) | Corn maize (intensive)  Sunflower (in/extensive)  Soy (intensive) | Corn maize (intensive)  Sunflower (in/extensive)  Soy (intensive) |
|  | Livestock capacity  (LU = livestock unit) | Small dairy farm: 0.8 LU/ha  Large dairy farm: 0.8 LU/ha  Suckler farm: 1.0 LU/ha | Small dairy farm: 0.5 LU/ha  Large dairy farm: 1.1 LU/ha  Suckler farm: 0.7 LU/ha | Large dairy farm: 1.1 LU/ha  Suckler farm: 1.5 LU/ha |
|  | Stall system | Free-range | Mixed | Kept only indoors |
|  | Milk yield | 5500 l/year | Same as the reference  (7000 l/year, 6500 l/year for grass-based fodder system*) | 8500 l/year |
|  | Milk price | 0.9 CHF/kg | 0.6 CHF/kg | 0.5 CHF/kg |
|  | Meat price | 13.5 CHF/kg (226 kg per cow) | 6.1 CHF/kg (250 kg per cow) | 5.4 CHF/kg (250 kg per cow) |
|  | Feed system  (5% stock loss of yields considered) | Only-grass-based fodder system/no concentrate | Same as the reference  (Small dairy farm/suckler farm with grass-based fodder system, large dairy farm = no restriction) | Only hay and concentrate |
|  | Pasture use | Minimum 20% of the required energy | Minimum 20% of the required energy | None |
|  | Lactation period | Longer | Same as the reference | Shorter |
|  | Biodiversity measure | Biodiversity measures at least 15% of the farmland and 10% of arable land | Higher than the reference (biodiversity measures at least 10% of the farmland and 4% of arable land) | None |
|  | Flower strip restriction | Less than 15% of arable land | Less than 5% of arable land | Less than 5% of arable land |

*The grass-based fodder system requires the share of concentrates should be less than 10% of the total feed in dry matter, and at least 75% of it should be grass fodder.

**Table S2.2** Relative parameter change for the modelled subsidies compared to the reference (100%) based on our assumptions, which are consistent with the storylines of the corresponding Eur-Agri-SSPs

| **Direct payment category** | **Item** | **Reference** | **SBL-Agri-SSP1 (Regional organic path)** | **SBL-Agri-SSP2 (Business-As-Usual path)** | **SBL-Agri-SSP5 (Liberalisation)** |
| --- | --- | --- | --- | --- | --- |
| DP1-Cultural landscapes | Cultivation contribution | 100% | 75% | 100% | 0% |
| DP2-Food security | Basic contri.to arable crops & ley | 100% | 75% | 100% | 0% |
|  | Basic contribution for non-EFA* | 100% | 75% | 100% | 0% |
|  | EFA on Grassland | 100% | 125% | 110% | 0% |
|  | Contribution to arable crops | 100% | 75% | 100% | 0% |
|  | Contribution to special cultures | 100% | 75% | 100% | 0% |
|  | Cereal allowance (price support) | 100% | 75% | 100% | 0% |
| DP3-Production system | Extensive contribution | 100% | 125% | 110% | 0% |
|  | GMF** contribution | 100% | 125% | 100% | 0% |
|  | Organic contribution | 100% | 125% | - | - |
| DP4-Biodiversity | Less intensive meadow | 100% | 110% | 105% | 0% |
|  | Extensive meadow | 100% | 125% | 110% | 0% |
|  | Extensive pasture | 100% | 125% | 110% | 0% |
|  | Payment for trees | 100% | 125% | 110% | 0% |
|  | Flower strips | 100% | 125% | 110% | 0% |

*EFA…Ecological focused area, **GMF…The grass-based fodder system

**Table S2.3** Relative change for variable cost calculation based on our assumptions, which are consistent with the storyline of the corresponding Eur-Agri-SSPs

| **Category** | **Item** | **Reference** | **SBL-Agri-SSP1 (Regional organic path)** | **SBL-Agri-SSP2 (Business-As-Usual path)** | **SBL-Agri-SSP5 (Liberalisation)** |
| --- | --- | --- | --- | --- | --- |
| Seeds | Seed processed | 100% | 100% | 100% | 100% |
|  | Seed unprocessed | 100% | 100% | 100% | 100% |
| Fertiliser | N | 100% | - | 100% | 100% |
|  | P_2_O_5_ | 100% | - | 100% | 100% |
|  | K_2_O | 100% | - | 100% | 100% |
|  | Mg | 100% | - | 100% | 100% |
|  | Organic supplementary fertiliser | 100% | 100% | 100% | 100% |
| Plant protection | Herbicide | 100% | - | 100% | 100% |
|  | Fungicide | 100% | - | 100% | 100% |
|  | Insecticide | 100% | - | 100% | 100% |
|  | Growth regulator | 100% | - | 100% | 100% |
|  | Trichogramma | 100% | - | 100% | 100% |
| Others | Hail insurance | 100% | 150% | 150% | 150% |
|  | Cleaning | 100% | 100% | 100% | 100% |
|  | Drying | 100% | 187% | 187% | 100% |
|  | Miscellaneous contributions | 100% | 100% | 100% | 100% |
|  | Contract work | 100% | 100% | 100% | 100% |
|  | Variable machine costs | 100% | 100% | 100% | 100% |
|  | Interest claim | 100% | 100% | 100% | 100% |
|  | Control and label costs | 100% | 100% | 100% | 100% |

**Table S2.4** Description of different orchard meadow management modelled in LUCIA

|  | Orchard meadows (type A) | Orchard meadows (type B) | Orchard meadows (type C) |
| --- | --- | --- | --- |
| Description | Maintaining the existing orchard trees for commercial cherry production | Maintaining the existing orchard trees to receive subsidies (no commercial cherry production) | Expanding orchard meadows (planting trees and maintaining over 10 years) |
| Number of trees | 60 ha^−1^ | 30 ha^−1^ | 30 ha^−1^ |
| Grassland management | Less intensive (for SBL-Agri-SSP1, Extensive) | Extensive | Extensive |
| Cherry yield | 50 kg/tree (38 CHF/kg for SBL-SSP1) | — | — |
| Price of cherry | 1.2 CHF/kg (3.8 CHF/kg for SBL-Agri-SSP1) | — | — |
| Future cost | — | — | Considered (over 10 years) |
| Constraint | Limit to the area, same as the observation | Limit to the area, same as the observation | No limitation |

# **Reference**

De Ponti, T., Rijk, B., & Van Ittersum, M. K. (2012). The crop yield gap between organic and conventional agriculture. *Agricultural Systems*, *108*, 1–9. https://doi.org/10.1016/j.agsy.2011.12.004

Mitter, H., Techen, A. K., Sinabell, F., Helming, K., Schmid, E., Bodirsky, B. L., Holman, I., Kok, K., Lehtonen, H., Leip, A., Le Mouël, C., Mathijs, E., Mehdi, B., Mittenzwei, K., Mora, O., Øistad, K., Øygarden, L., Priess, J. A., Reidsma, P., … Schönhart, M. (2020). Shared Socio-economic Pathways for European agriculture and food systems: The Eur-Agri-SSPs. *Global Environmental Change*, *65*(December 2019), 102159. https://doi.org/10.1016/j.gloenvcha.2020.102159
