# Supplementary material for: Towards diverse agricultural land uses: socio-ecological implications of European agricultural pathways for a Swiss orchard region
Source: Reg Environ Change. 2023 Jul 22;23(3):97. doi: 10.1007/s10113-023-02092-5 (PMC10363045; doi:10.1007/s10113-023-02092-5)
Supplement: Supplementary file 3 — Supplementary file3 (DOCX 32 KB) [file 10113_2023_2092_MOESM3_ESM.docx]

*Regional Environmental Change*

**Online Resource 3 (Gross margins of modelled farm activities)**

Towards diverse agricultural land uses: socio-ecological implications of European agricultural pathways for a Swiss orchard region

Takamasa Nishizawa^*^, Sonja Kay, Johannes Schuler, Noëlle Klein, Tobias Conradt, Michael Mielewczik, Peter Zander, Joachim Aurbacher, Felix Herzog

*Corresponding author: Takamasa Nishizawa, Leibniz Centre for Agricultural Landscape Research (ZALF) e.V., Farm Economics and Ecosystem Services, Müncheberg, Germany

E-Mail: [takamasa.nishizawa@zalf.de](mailto:takamasa.nishizawa@zalf.de); Tel.: +49 (0)33432 82-490; Fax: +49 (0)33432 82-4082

**Table S4.1** Gross margin (CHF/ha) with and without subsidies over farm activities and different intensities

|  |  |  |  | **Reference** | | **SBL-Agri-SSP1** | | **SBl-Agri-SSP2** | | **SBL-Agri-SSP5** | |
| --- | --- | --- | --- | --- | --- | --- | --- | --- | --- | --- | --- |
| **Land use** | **Production category** | **Intensity/ management** | **Farm activity** | **GM no subsidy** | **GM with subsidy** | **GM no subsidy** | **GM with subsidy** | **GM no subsidy** | **GM with subsidy** | **GM no subsidy** | **GM with subsidy** |
| Grassland | Fodder production | Intensive | Meadow | -900 | 300 | -965 | 35 | -938 | 262 | -938 | - |
|  | *internal use |  | Pasture | -476 | 724 | -537 | 463 | -514 | 686 | -514 | - |
|  |  | Less intensive | Meadow | -460 | 740 | -503 | 879 | -498 | 770 | -498 | - |
|  |  | Extensive | Meadow | -172 | 1438 | -172 | 1791 | -172 | 1569 | -172 | - |
|  |  |  | Pasture | -79 | 1121 | -79 | 921 | -79 | 1121 | -79 | - |
|  |  | Less intensive | Orchard meadow Type A | -6945 | -2745 | 482 | 5390 | -6945 | -2423 | -7305 | - |
|  |  | Extensive | Orchard meadow Type B | -172 | 2938 | -172 | 3441 | -172 | 3174 | -172 | - |
|  |  | Extensive | Orchard meadow Type C | -897 | 1013 | -897 | 1216 | -897 | 1129 | -897 | - |
| Grassland | Fodder production | Intensive | Meadow | 2090 | 3290 | 864 | 1864 | 1803 | 3003 | 1491 | - |
|  | *to sell |  | Pasture | 2054 | 3254 | 1022 | 2022 | 1821 | 3021 | 1547 | - |
|  |  | Less intensive | Meadow | 1012 | 2212 | 397 | 1780 | 851 | 2592 | 698 | - |
|  |  | Extensive | Meadow | 530 | 2140 | 257 | 2220 | 471 | 2212 | 398 | - |
|  |  |  | Pasture | 473 | 1673 | 261 | 1711 | 431 | 1631 | 371 | - |
|  |  | Less intensive | Orchard meadow Type A | -5694 | -2994 | 871 | 5779 | -5799 | -1276 | -6289 | - |
|  |  | Extensive | Orchard meadow Type B | 460 | 3570 | 214 | 3827 | 407 | 3753 | 341 | - |
|  |  | Extensive | Orchard meadow Type C | -266 | 1644 | -511 | 1602 | -318 | 1708 | -384 | - |

|  |  |  |  | **Reference** | | **SBL-Agri-SSP1** | | **SBl-Agri-SSP2** | | **SBL-Agri-SSP5** | |
| --- | --- | --- | --- | --- | --- | --- | --- | --- | --- | --- | --- |
| **Land use** | **Production category** | **Intensity/ management** | **Farm activity** | **GM no subsidy** | **GM with subsidy** | **GM no subsidy** | **GM with subsidy** | **GM no subsidy** | **GM with subsidy** | **GM no subsidy** | **GM with subsidy** |
| Arable land | Fodder production | Intensive | Fodder wheat | -1408 | 112 | - | - | -1518 | 2 | -1476 | - |
|  | *internal use |  | Triticale | -1277 | 243 | - | - | -1391 | 129 | -1350 | - |
|  |  |  | Winter barley | -1384 | 136 | - | - | -1502 | 18 | -1450 | - |
|  |  |  | Lay pasture | -1289 | -89 | - | - | -1359 | -159 | -1359 | - |
|  |  |  | Soy | -1622 | 778 | - | - | -1731 | 669 | -1646 | - |
|  |  | Extensive | Fodder wheat | -1277 | 643 | - | - | -1366 | 594 | -1332 | - |
|  |  |  | Triticale | -1210 | 710 | - | - | -1299 | 661 | -1266 | - |
|  |  |  | Winter barley | -1292 | 628 | - | - | -1387 | 573 | -1345 | - |
|  |  |  | Lay pasture | -1182 | 18 | - | - | -1252 | -52 | -1252 | - |
|  |  |  | Silo-green corn | -1367 | 33 | - | - | -1414 | -14 | -1414 | - |
|  |  | Organic | Clover grass | - | - | -1096 | 154 | - | - | - | - |
| Arable land | Fodder production | Intensive | Fodder wheat | 1504 | 3024 | - | - | 1277 | 2797 | 982 | - |
|  | *to sell |  | Triticale | 1483 | 3003 | - | - | 1230 | 2750 | 939 | - |
|  |  |  | Winter barley | 1595 | 3115 | - | - | 1340 | 2860 | 1061 | - |
|  |  |  | Lay pasture | 1321 | 2521 | - | - | 1033 | 2233 | 761 | - |
|  |  |  | Soy | -488 | 1912 | - | - | -649 | 1751 | -580 | - |
|  |  | Extensive | Fodder wheat | 1089 | 3009 | - | - | 904 | 2864 | 665 | - |
|  |  |  | Triticale | 959 | 2879 | - | - | 760 | 2720 | 532 | - |
|  |  |  | Winter barley | 1129 | 3049 | - | - | 922 | 2882 | 695 | - |
|  |  |  | Lay pasture | 978 | 2178 | - | - | 728 | 1928 | 503 | - |
|  |  |  | Silo-green corn | 1119 | 2519 | - | - | 1021 | 2421 | 753 | - |
| Arable land | Crops for human | Intensive | Spelt wheat | 877 | 2397 | - | - | 830 | 2350 | 536 | - |
|  |  |  | Winter wheat | 1783 | 3303 | - | - | 1706 | 3226 | 1301 | - |
|  |  |  | Oats | 679 | 2199 | - | - | 561 | 2081 | 351 | - |
|  |  |  | Corn maize | 1723 | 3123 | - | - | 1456 | 2856 | 1101 | - |
|  |  |  | Sunflower | 1047 | 3147 | - | - | 850 | 2950 | 619 | - |
|  |  | Extensive | Spelt wheat | 1146 | 3066 | - | - | 1107 | 3067 | 781 | - |
|  |  |  | Winter wheat | 1229 | 3149 | - | - | 1168 | 3128 | 850 | - |
|  |  |  | Oats | 403 | 2323 | - | - | 304 | 2264 | 129 | - |
|  |  |  | Rye | 1240 | 3160 | - | - | 1224 | 3184 | 843 | - |
|  |  |  | Sunflower | 774 | 3274 | - | - | 609 | 3149 | 417 | - |
|  |  | Organic | Spelt wheat | - | - | 948 | 4088 | - | - | - | - |
|  |  |  | Winter wheat | - | - | 1758 | 4898 | - | - | - | - |
|  |  |  | Summer wheat | - | - | 1146 | 4286 | - | - | - | - |
|  |  |  | Corn maize | - | - | 2391 | 4941 | - | - | - | - |
|  |  |  | Sunflower | - | - | 803 | 4378 | - | - | - | - |
|  |  |  | Soy | - | - | 313 | 3613 | - | - | - | - |
|  |  |  | Flower strips | -1298 | 1202 | -1292 | 1833 | -1292 | 1458 | -1292 | - |
