# Supplementary material for: Towards diverse agricultural land uses: socio-ecological implications of European agricultural pathways for a Swiss orchard region
Source: Reg Environ Change. 2023 Jul 22;23(3):97. doi: 10.1007/s10113-023-02092-5 (PMC10363045; doi:10.1007/s10113-023-02092-5)
Supplement: Supplementary file 4 — Supplementary file4 (DOCX 40 KB) [file 10113_2023_2092_MOESM4_ESM.docx]

*Regional Environmental Change*

**Online Resource 4 (Supplementary results)**

Towards diverse agricultural land uses: socio-ecological implications of European agricultural pathways for a Swiss orchard region

Takamasa Nishizawa^*^, Sonja Kay, Johannes Schuler, Noëlle Klein, Tobias Conradt, Michael Mielewczik, Peter Zander, Joachim Aurbacher, Felix Herzog

*Corresponding author: Takamasa Nishizawa, Leibniz Centre for Agricultural Landscape Research (ZALF) e.V., Farm Economics and Ecosystem Services, Müncheberg, Germany

E-Mail: [takamasa.nishizawa@zalf.de](mailto:takamasa.nishizawa@zalf.de); Tel.: +49 (0)33432 82-490; Fax: +49 (0)33432 82-4082

**Table S4.1** Modelling outcomes of each farm activity over different intensities at the reference and under SBL-Agri-SSPs (unit: ha)

| **Land use** | **Intensity** | **Crops** | **EFA*** | **Ref** | **SBL-Agri-SSP1** | **SBL-Agri-SSP2** | **SBL-Agri-SSP5** |
| --- | --- | --- | --- | --- | --- | --- | --- |
| Grassland |  | Intensive meadow |  | 0 | 181 | 110 | 324 |
|  |  | Intensive pasture |  | 110 | 109 | 104 | 0 |
|  |  | Less intensive meadow | X | 0 | 0 | 0 | 0 |
|  |  | Extensive meadow | X | 0 | 0 | 62 | 0 |
|  |  | Extensive pasture | X | 0 | 0 | 0 | 0 |
|  |  | Orchard meadow Type A | X | 0 | 514 | 0 | 0 |
|  |  | Orchard meadow Type B | X | 852 | 325 | 819 | 0 |
|  |  | Orchard meadow Type C | X | 0 | 0 | 0 | 0 |
| Arable land | Intensive | Fodder wheat |  | 0 | 0 | 0 | 0 |
| (Fodder production) |  | Triticale |  | 0 | 0 | 0 | 0 |
|  |  | Winter Barley |  | 32 | 0 | 0 | 0 |
|  |  | Ley |  | 129 | 0 | 0 | 186 |
|  |  | Soy |  | — | 0 | 0 | 0 |
|  | Extensive | Fodder wheat |  | 0 | 0 | 3 | 0 |
|  |  | Triticale |  | 12 | 0 | 0 | 0 |
|  |  | Winter Barley |  | 0 | 0 | 11 | 0 |
|  |  | Ley pasture |  | 0 | 0 | 0 | 0 |
|  |  | Silo-green maize |  | 151 | 0 | 38 | 207 |
|  |  | Clover |  | — | 92 | 0 | — |
| Cash crops | Intensive | Spelt wheat |  | 0 | 0 | 101 | 0 |
| (cash production) |  | Winter Wheat |  | 411 | 0 | 0 | 728 |
|  |  | Oats |  | 0 | 0 | 263 | 0 |
|  |  | Corn |  | — | 0 | 0 | 335 |
|  |  | Sunflower |  | — | 0 | 0 | 0 |
|  | Extensive | Spelt wheat |  | 0 | 0 | 38 | 0 |
|  |  | Winter Wheat |  | 0 | 0 | 0 | 0 |
|  |  | Oats |  | 87 | 0 | 35 | 0 |
|  |  | Sunflower |  | — | 0 | 0 | 0 |
|  | Organic | Spelt wheat |  | — | 0 | — | — |
|  |  | Winter Wheat |  | — | 96 | — | — |
|  |  | Oats |  | — | 0 | — | — |
|  |  | Corn |  | — | 261 | — | — |
|  |  | Sunflower |  | — | 0 | — | — |
|  |  | Soy |  | — | 86 | — | — |
|  |  | Flower strips | X | 1 | 117 | 47 | 0 |
|  |  | Sum |  | 1784 | 1780 | 1784 | 1780 |

*EFA…Ecological focused area

**Table S4.2** Resulting socioeconomic and ecological indicators calculated based on the land use changes across SBL-Agri-SSPs simulated with LUCIA at the regional level and across farm types in comparison to the reference (Ref). The regional result is the area-weighted average over all farm types in the case study region. TGM: total gross margin. EFA: ecological focused area

| Region | Unit | Ref | SBL-Agri-SSP1 | SBL-Agri-SSP2 | SBL-Agri-SSP5 |
| --- | --- | --- | --- | --- | --- |
| TGM | CHF/ha | 4,284 | 5,337 | 4,753 | 2,826 |
| Subsidy | CHF/ha | 2,197 | 2,790 | 2,451 | 0 |
| TGM per labour hour | CHF/hour | 114 | 119 | 131 | 57 |
| N fertiliser | Kg/ha farmland | 68 | 65 | 81 | 148 |
| Plant protections | Times/ha arable land | 2.1 | 0.0 | 1.9 | 2.2 |
| Livestock intensity | LU/ha farmland | 0.9 | 0.9 | 0.8 | 1.1 |
| Cereal production | Kg | 34,424 | 26,771 | 30,077 | 85,005 |
| Milk production | 1,000 liter | 37,273 | 17,600 | 33,395 | 49116 |
| Beef production | 1,000 kg | 98 | 123 | 101 | 217 |
| Cherry production | 1,000 kg | 0 | 1,202 | 0 | 0 |
| Biodiversity score | Points/ha | 10.3 | 11.7 | 11.1 | 6.1 |
| EFA | % of farmland | 48% | 54% | 51% | 0% |
| EFA on arable land | % of arable land | 0% | 16% | 4% | 0% |
| Trees (total) | Number/region | 25,572 | 40,583 | 24,575 | 0 |
| Small dairy farm | Unit | Ref | SBL-Agri-SSP1 | SBL-Agri-SSP2 | SBL-Agri-SSP5 |
| TGM | CHF/ha | 4,716 | 7,254 | 4,717 | - |
| Subsidy | CHF/ha | 2,362 | 2,853 | 2,602 | - |
| TGM per labour hour | CHF/hour | 100 | 102 | 118 | - |
| N fertiliser | Kg/ha farmland | 70 | 79 | 69 | - |
| Plant protection | Times/ha arable land | 2.0 | 0.0 | 2.2 | - |
| Livestock intensity | LU/ha farmland | 0.6 | 0.8 | 0.5 | - |
| Cereal production | Kg | 513 | 338 | 629 | - |
| Milk production | 1,000 liter | 61 | 73 | 56 | - |
| Beef production | Kg | 0 | 0 | 0 | - |
| Cherry production | 1,000 kg | 0 | 20 | 0 | - |
| Biodiversity score | Points/ha | 10.0 | 11.1 | 10.3 | - |
| EFA | % | 43% | 47% | 45% | - |
| EFA on arable land | % of arable land | 0 | 10% | 4% | - |
| Trees | Number/farm | 257 | 516 | 284 | - |
| Large dairy farm | Unit | Ref | SBL-Agri-SSP1 | SBL-Agri-SSP2 | SBL-Agri-SSP5 |
| TGM | CHF/ha | 5,821 | 7,049 | 5,838 | 3,261 |
| Subsidy | CHF/ha | 1,910 | 2,607 | 1,959 | 0 |
| TGM per labour hour | CHF/hour | 84 | 100 | 89 | 88 |
| N fertiliser | Kg/ha farmland | 82 | 87 | 108 | 127 |
| Plant protection | Times/ha arable land | 1.9 | 0.0 | 2.1 | 2.4 |
| Livestock intensity | LU/ha farmland | 1.1 | 0.8 | 1.1 | 0.8 |
| Cereal production | Kg | 767 | 678 | 781 | 4,281 |
| Milk production | 1,000 liter | 218 | 128 | 263 | 379 379 |
| Beef production | Kg | 0 | 0 | 0 | 0 |
| Cherry production | 1,000 kg | 0 | 31 | 0 | 0 |
| Biodiversity score | Points/ha | 9.5 | 10.7 | 9.8 | 5.7 |
| EFA | % | 38% | 42% | 32% | 0% |
| EFA on arable land | % of arable land | 0% | 10% | 4% | 0% |
| Trees | Number/farm | 464 | 798 | 401 | 0 |
|  |  |  |  |  |  |
|  |  |  |  |  |  |
|  |  |  |  |  |  |
|  |  |  |  |  |  |
|  |  |  |  |  |  |
|  |  |  |  |  |  |
|  |  |  |  |  |  |
|  |  |  |  |  |  |
|  |  |  |  |  |  |
|  |  |  |  |  |  |
|  |  |  |  |  |  |
|  |  |  |  |  |  |
|  |  |  |  |  |  |
|  |  |  |  |  |  |
| Suckler farm | Unit | Ref | SBL-Agri-SSP1 | SBL-Agri-SSP2 | SBL-Agri-SSP5 |
| TGM | CHF/ha | 3,588 | 5,665 | 3,863 | 1,834 |
| Subsidy | CHF/ha | 2,316 | 2,639 | 2,658 | 0 |
| TGM per labour hour | CHF/hour | 112 | 126 | 137 | 53 |
| N fertiliser | Kg/ha farmland | 62 | 95 | 68 | 182 |
| Plant protection | Times/ha arable land | 1.9 | 0.0 | 2.2 | 1.9 |
| Livestock intensity | LU/ha farmland | 0.8 | 1.0 | 0.7 | 1.5 |
| Cereal production | Kg | 691 | 242 | 725 | 1,458 |
| Milk production | 1,000 liter | 0.0 | 0.0 | 0.0 | 0.0 |
| Beef production | Kg | 5,652 | 5,870 | 5,326 | 15,625 |
| Cherry production | 1,000 kg | 0 | 36 | 0 | 0 |
| Biodiversity score | Points/ha | 10.7 | 12.1 | 11.1 | 6.0 |
| EFA | % | 51% | 53% | 53% | 0% |
| EFA on arable land | % of arable land | 0% | 10% | 4% | 0% |
| Trees | Number/farm | 514 | 918 | 536 | 0 |
| Orchard farm | Unit | Ref | SBL-Agri-SSP1 | SBL-Agri-SSP2 | SBL-Agri-SSP5 |
| TGM | CHF /ha | 3,200 | 3,469 | 3,215 | 1,197 |
| Subsidy | CHF/ha | 2,352 | 2,982 | 2,676 | 0 |
| TGM per labour hour | CHF/hour | 153 | 134 | 175 | 99 |
| N fertiliser | Kg/ha farmland | 65 | 18 | 19 | 122 |
| Plant protection | Times/ha arable land | 2.5 | 0.0 | 1.0 | 2.0 |
| Cereal production | Kg | 482 | 719 | 369 | 2,979 |
| Cherry production | 1,000 kg | 0 | 0 | 0 | 0 |
| Biodiversity score | Points/ha | 11.1 | 12.7 | 12.5 | 5.3 |
| EFA | % | 61% | 71% | 73% | 0% |
| EFA on arable land | % of arable land | 0% | 25% | 4% | 0% |
| Trees | Number/farm | 637 | 732 | 769 | 0 |
| Small farm | Unit | Ref | SBL-Agri-SSP1 | SBL-Agri-SSP2 | SBL-Agri-SSP5 |
| TGM | CHF /ha | 3,174 | 3,774 | 3,063 | - |
| Subsidy | CHF/ha | 2,238 | 3,007 | 2,564 | - |
| TGM per labour hour | CHF/hour | 160 | 145 | 179 | - |
| N fertiliser | kg/ha farmland | 51 | 35 | 22 | - |
| Plant protection | Times/ha arable land | 2.5 | 0.0 | 1.0 | - |
| Cereal production | Kg | 142 | 184 | 20 | - |
| Cherry production | 1,000 kg | 0 | 0 | 0 | - |
| Biodiversity score | Points/ha | 10.3 | 10.5 | 12.4 | - |
| EFA | % | 53% | 44% | 72% | - |
| EFA on arable land | % of arable land | 0% | 25% | 4% | - |
| Trees | Number/farm | 135 | 63 | 135 | - |
